# Bergmann Glia and the Recognition Molecule CHL1 Organize GABAergic Axons and Direct Innervation of Purkinje Cell Dendrites

**DOI:** 10.1371/journal.pbio.0060103

**Published:** 2008-04-29

**Authors:** Fabrice Ango, Caizhi Wu, Johannes J. Van der Want, Priscilla Wu, Melitta Schachner, Z. Josh Huang

**Affiliations:** 1 Cold Spring Harbor Laboratory, Cold Spring Harbor, New York, United States of America; 2 CNRS, UMR 5203, Institut de Génomique fonctionnelle, INSERM, U661, Montpellier, France; 3 Université Montpellier 1 and 2, Montpellier, France; 4 Department of Cell Biology, Laboratory for Electron Microscopy, University Medical Center Groningen, University of Groningen, Groningen, The Netherlands; 5 Zentrum fur Molekulare Neurobiologie, Universitat Hamburg, Hamburg, Germany; 6 Keck Center for Collaborative Neuroscience, Department of Cell Biology, Rutgers University, Piscataway, New Jersey, United States of America; University of California, San Diego, United States of America

## Abstract

The geometric and subcellular organization of axon arbors distributes and regulates electrical signaling in neurons and networks, but the underlying mechanisms have remained elusive. In rodent cerebellar cortex, stellate interneurons elaborate characteristic axon arbors that selectively innervate Purkinje cell dendrites and likely regulate dendritic integration. We used GFP BAC transgenic reporter mice to examine the cellular processes and molecular mechanisms underlying the development of stellate cell axons and their innervation pattern. We show that stellate axons are organized and guided towards Purkinje cell dendrites by an intermediate scaffold of Bergmann glial (BG) fibers. The L1 family immunoglobulin protein Close Homologue of L1 (CHL1) is localized to apical BG fibers and stellate cells during the development of stellate axon arbors. In the absence of CHL1, stellate axons deviate from BG fibers and show aberrant branching and orientation. Furthermore, synapse formation between aberrant stellate axons and Purkinje dendrites is reduced and cannot be maintained, leading to progressive atrophy of axon terminals. These results establish BG fibers as a guiding scaffold and CHL1 a molecular signal in the organization of stellate axon arbors and in directing their dendritic innervation.

## Author Summary

Large principal neurons in vertebrate neural circuits often consist of distinct anatomical and physiological compartments, which allow distributed and compartmentalized signaling and greatly increase the computational power of single neurons. Superimposed upon this intrinsic compartmental architecture is the subcellular organization of synaptic inputs, which exert local control over the biophysical properties and differentially regulate the input, integration, and output of principal neurons. In the cerebellar cortex, Purkinje neurons are innervated by GABA inhibitory synapses from the stellate and basket cells at dendrites and soma-axon initial (AIS) segments, respectively. Previous studies have shown that an L1 family immunoglobulin cell adhesion molecule (neurofascin186) is distributed as a subcellular gradient and directs basket cell axons to innervate Purkinje cell AIS. Here, we examine the mechanisms underlying the innervation of Purkinje cell dendrites by stellate axons. We found that stellate axons are organized into characteristic trajectories and guided towards Purkinje dendrites by an intermediate scaffold of astroglia—the Bergmann glial (BG) fibers. Another member of L1 family, Close Homologue of L1 (CHL1), is localized to BG fibers and stellate cells, and contributes to the organization of stellate axons along BG fibers and to the innervation of Purkinje cell dendrites.

## Abstract 3

Subcellular synapse organization regulates the input, integration, and output of target neurons. An astroglial scaffold and an L1 family cell adhesion molecule contribute to dendritic innervation by GABA inhibitory synapses.

## Introduction

Neurons are often characterized by striking polarity and extensive subcellular specialization. For example, large principal neurons in many vertebrate neural circuits consist of distinct anatomical and physiological compartments [1], which allow distributed and compartmentalized signaling [2–4], and may greatly increase the computation power of single neurons [5]. Indeed, the biophysical and signaling machineries of principal neurons are organized into discrete subcellular domains [6], best exemplified by the highly restricted distribution of all major classes of ion channels along the axon–dendritic surface [7]. Superimposed upon the intrinsic compartmental architecture of principal neurons is the subcellular organization of synaptic inputs [8,9], which exert further control over the biophysical properties, not only within a neuron, but also within a neural ensemble [10]. Subcellular synapse organization is a prominent feature of neuronal wiring specificity, but the underlying cellular and molecular mechanisms are not well understood.

A prime example of subcellular synapse organization is the Purkinje neurons of the cerebellum. The cerebellar cortex is organized as a near lattice-like circuit architecture along the two axes of the cerebellar lobules, the translobular and parlobular planes [11]. At the focal position in the cerebellar cortex and as its sole output, Purkinje neurons are restricted in the translobular plane and receive at least four sets of subcellularly targeted excitatory and inhibitory inputs [11]. The glutamatergic parallel fibers synapse onto the slender spines of the more distal dendrites, whereas the climbing fibers prefer the stubby spines of the proximal dendrite. In addition, the GABAergic basket interneurons target Purkinje cell soma and axon initial segment (AIS), whereas the stellate interneurons innervate the dendritic shafts. The mechanisms underlying subcellular synapse organization along Purkinje neurons are only beginning to be understood [12]. There is evidence that the innervation of Purkinje AIS by basket interneurons is guided by a subcellular gradient of neurofascin186, an L1 family immunoglobulin cell adhesion molecule, recruited by the ankyrinG membrane adaptor protein [13]. On the other hand, the mechanisms that direct the innervation of Purkinje dendrites by stellate interneurons are unknown.

Stellate cells mainly occupy the upper half of the molecular layer (ML) and are the only cell type of the upper third of the ML. Like the basket cells, stellate cells extend their axons within the translobular (e.g., parasagittal) plane of the cerebellar cortex [11]. Although these axon arbors range from relatively simple to fairly complex, the most characteristic feature is their largely vertically oriented ascending and descending collaterals, which innervate multiple Purkinje dendrites along their path [11]. Unlike climbing fibers, which have been well documented to grow along and eventually innervate single Purkinje dendrites, the cellular and developmental process by which stellate axon approach and innervate Purkinje dendrites have not been described.

Bergmann glia (BG) cells are highly polarized astrocytes, whose radial fibers dominate the cerebellar cortex [11,14,15]. During postnatal cerebellar development, the apical BG fibers form the earliest radial structures across the cerebellar cortex [14]; BG fibers subsequently undergo dramatic differentiation and are transformed into a highly elaborate meshwork, dominated by a scaffold of radial fibers [16–18]. The Bergmann glia cells are positioned to interact with multiple neuronal components and likely contribute to multiple aspects of cerebellar circuit assembly at different developmental stages. Whether BG fibers play a role in axon guidance and organization at later stages, in addition to guiding granule cell migration [19], has not been explored. By using a genetic strategy to simultaneously label stellate axons and BG fibers at high resolution, here we provide evidence that BG fibers constitute an intermediate template in the organization of stellate axon arbors into characteristic trajectories, and in their guidance to innervate Purkinje dendrites.

The L1 family immunoglobulin cell adhesion molecules (L1CAMs) have been implicated in axon growth, guidance [20], and the subcellular organization of GABAergic synapses [12]. Given the role of NF186 in the targeting of basket axon and pinceau synapses to Purkinje AIS [13], here we explored whether other members of the L1CAMs might contribute to the organization of stellate axons and innervation of Purkinje dendrites. We found that each member of the L1 family is localized to subcellular domains in neurons and glia in the developing and mature cerebellar cortex. In particular, CHL1 (Close Homologue of L1) is prominently expressed on BG fibers during the development of stellate axons and their innervation. In addition, we demonstrate a crucial and highly specific role of CHL1 in the patterning of stellate axons and in targeting their innervation to Purkinje cell dendrites.

## Results

### Stellate and Basket Axons Use Different Cellular Mechanisms to Innervate Subcellular Domains of Purkinje Neurons

**Figure 1:**
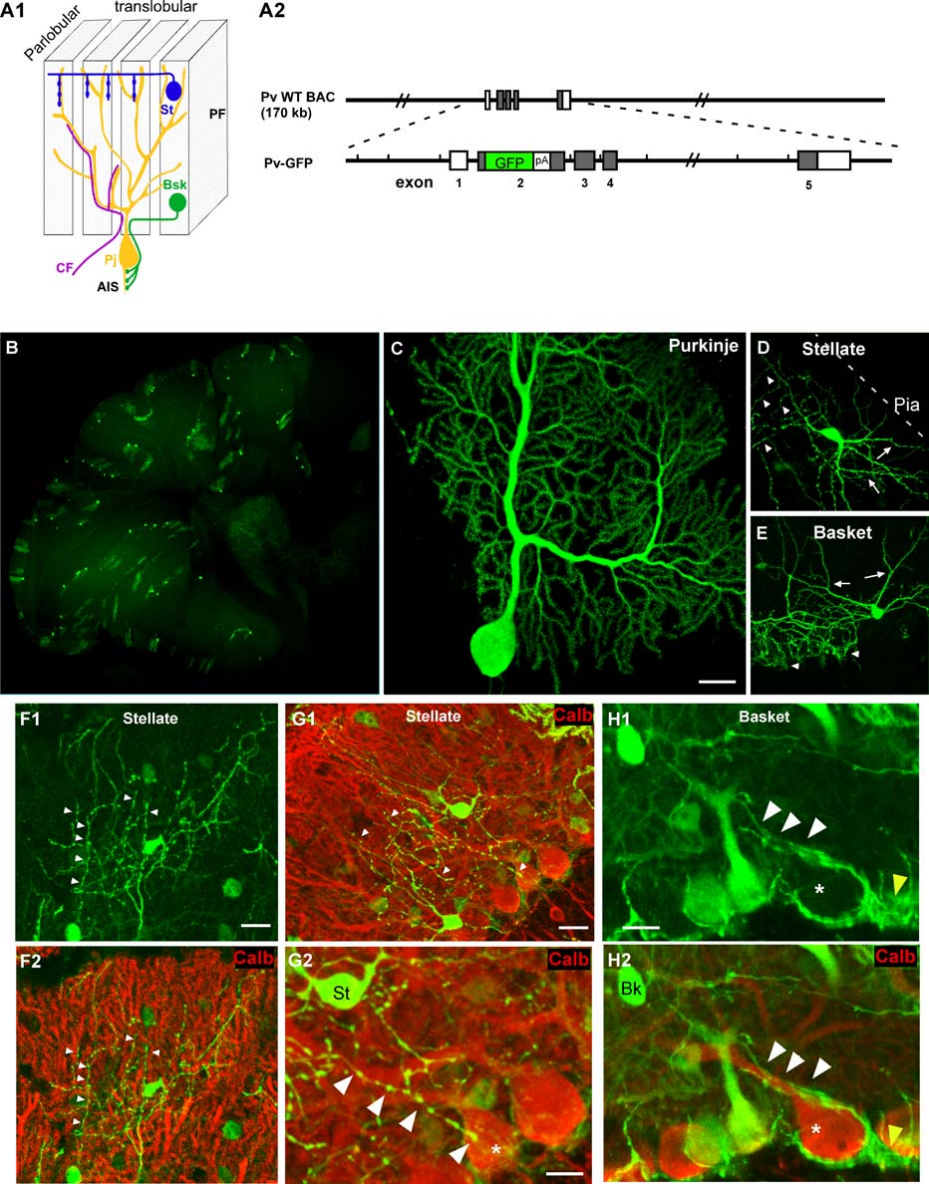
Stellate and Basket Cells Use Different Cellular Mechanisms to Innervate Purkinje Cells (A1) A schematic of the major neuronal components of the cerebellar cortex organized along the translobular and parlobular plane. Bsk, basket cell; CF, climbing fiber; PF, parallel fiber; Pj, Purkinje cell; St, stellate cell. (A2) A schematic of the PV-GFP BAC reporter construct. Long black lines indicate the PV BAC clone; shaded boxes, coding exons; open boxes, noncoding exons. Sequences coding for EGFP and polyadenylation signal (pA) were inserted at the translation initiation site (see Materials and Methods). (B) Low-magnification view of a cerebellar sagital section from an adult PV-GFP reporter mouse (B20 line). Note the low density of cells expressing GFP. (C–H) Individual Purkinje (C), stellate (D), and basket cells (E) can be resolved with synaptic resolution to their entirety. Arrows in (D) and (E) indicate dendrites. The basket axons ([E] and [H], arrowheads) are smooth, grow along Purkinje proximal dendrite labeled by calbindin (H2), soma (star), AIS, and form pinceau synapses (yellow arrowheads) at AIS. The stellate axons ([D], [F], and [G], arrowheads) are beaded, send ascending and descending collaterals that intercept with Purkinje dendrites ([F2] and [G2], labeled by calbindin immunofluorescence) at sharp angles. (G2) is a higher magnification of (G1). Note a straight and descending stellate axon branch (arrowheads) that reaches a Purkinje cell soma (star) but, in contrast to basket axons, does not grow along the Purkinje cell and terminates abruptly. Bk, basket cell; St, stellate cell. Scale bars indicate 20 μm.

To investigate the cellular mechanisms underlying development of the stellate axon arbor and innervation pattern, it is necessary to visualize stellate axons together with their postsynaptic targets at high resolution and during the developmental process. We generated several lines of bacterial artificial chromosome (BAC) transgenic reporter mice to achieve such visualization. The calcium binding protein parvalbumin (Pv) is normally expressed in all Purkinje, basket, and stellate neurons in the postnatal cerebellar cortex. In our Pv-GFP BAC transgenic mice (Figure 1A), different fractions of Purkinje, basket, and stellate neurons express green fluorescent protein (GFP) among different stable lines (from a few percent to near 100%; unpublished data), likely due to different genomic integration sites of the transgene. In the B20 line, sparse GFP expression allowed visualization of single Purkinje, basket, and stellate cells with synaptic resolution (Figure 1B–1E). Combined with generic Purkinje cell markers (e.g., calbindin), we were able to examine the precise trajectory of stellate and basket axons, and compare how they approach and innervate postsynaptic Purkinje neurons.

Stellate and basket cells occupy mainly the upper or lower half of the ML, respectively. Consistent with previous Golgi studies [11], GFP labeling revealed distinct features of stellate and basket axons in their morphology and subcellular innervation pattern, even when they are found at the same location in the mid-lower ML (Figure 1F–1H). For example, basket axons were smooth, whereas stellate axons were beaded. Although basket axons extended terminal branches along Purkinje cell proximal dendrites, soma, and towards AISs (Figure 1H), stellate axons clearly did not extend along the distinct contours of Purkinje dendrites. Instead, stellate axon arbors were often characterized by rather straight ascending and descending collaterals (also described in [11]) that crossed Purkinje cell dendrites at rather sharp angles (Figure 1F and 1G). Interestingly, the axon tips of stellate cells in the lower ML often reached the Purkinje cell layer (PCL), as the basket axons. However, these descending collaterals never extended along Purkinje dendrite-soma-AIS, but always had rather straight paths that terminated abruptly at the PCL (Figure 1G). These contrasting features suggest that basket and stellate axons approach and innervate Purkinje neurons via profoundly different cellular and developmental mechanisms. Furthermore, the arborization and innervation patterns of stellate axons also contrast those of a dendrite-targeting climbing fiber, which grows along and “monopolizes” an entire Purkinje dendrite [11]. These comparisons raise an obvious question: how do stellate axons approach and innervate segments of multiple Purkinje dendrites without growing along any single target?

### Development of Stellate Axon Arbor and Dendritic Innervation

**Figure 2:**
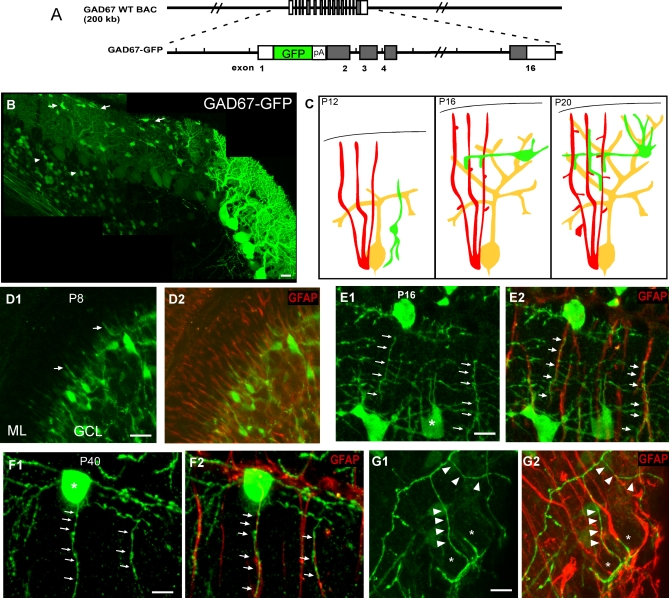
Developing Stellate Axons Extend along Bergmann Glial Fibers (A) A schematic of the GAD67-GFP BAC reporter construct. Long black lines indicate the GAD67 BAC clone; shaded boxes, coding exons; open boxes, noncoding exons. Sequences coding for EGFP and polyadenylation signal (pA) were inserted at the translation initiation site. (B) During the second postnatal week, stellate neurons migrate into the ML, reaching their laminar position between P12 and P16. At P14, some stellate cells already settled in the upper ML close to the pia (arrows), while other interneurons were still migrating in the white matter (arrowheads). (C) Schematic representations of the development of stellate axons (green) and their relationship to Purkinje cells (yellow) and Bergmann glia (red) between P12 and P20. (D) At P8, BG fibers (GFAP immunofluorescence in [D2]) were already prominent in the ML while interneurons were migrating towards and into the ML ([D1], arrows indicate neurites). (E) At P16, stellate cells (E1) extended their axons with ascending and descending branches that strictly adhered to the GFAP-labeled BG fibers (E2, arrows). (F) At more-mature ages (P40), stellate cell axons (F1) were still associated with BG fibers (F2). (G) Basket axons at P40 (G1, arrowheads) did not associate with GFAP-labeled BG fibers (G2) but outlined Purkinje cell soma (stars). Scale bars indicate 20 μm

To better understand the developmental process by which stellate axons innervate Purkinje dendrites, we used our reporter mice expressing GFP from the GAD67 promoter elements (GAD67-GFP BAC reporter mice, Figure 2A and [13]), which label Purkinje, basket, and stellate cells from embryonic stage to adulthood. In the G42 line, both interneurons and Purkinje cells were labeled; this line was mainly used to characterize the migration of stellate cells in the first two postnatal weeks (summarized in Figure 2C). In the G1 line, GFP was mainly expressed by basket and stellate cells; this line was used to characterize the development of stellate axons.

Basket and stellate cells are derived from dividing progenitors in the postnatal cerebellar white matter (WM; [21]). These progenitors migrate into the cerebellar cortex in the first two postnatal weeks as simple unipolar cells until arriving in the ML. They then undergo a series of morphological transformations that culminate in formation of mature interneurons during the third and fourth postnatal week. However, the morphological maturation of stellate axons has not been well described. Compared to basket cells, stellate cell precursors migrate into the ML a few days later, peaking between postnatal day 8 and 11 (P8–P11) but continue to arrive as late as P14 (Figure 2B–2D; also see [22]). Using our G1 reporter mice, we found that upon reaching their positions in the ML, stellate cells first appeared bipolar and extended largely horizontally oriented neurites (Figure 2B; also see [21]). Between P16–18, stellate axons sent ascending and descending collaterals (Figure 2E), which further gave rise to plexus of more elaborate branches in the subsequent 2 wk (Figures 2F). Mature stellate axon arbors range from relatively simple to fairly complex; the most characteristic feature is their largely vertically oriented ascending and descending collaterals [11]. Our GFP labeling is highly consistent with these previous descriptions using Golgi methods (Figure 2E and 2F, and unpublished data). The stereotyped morphology and development of stellate axons pose an obvious question: how are they organized into characteristic trajectories, presumably by mechanisms other than Purkinje cell dendrites?

### Close Association of Stellate Axons with Bergmann Glial Fibers during the Development of Dendritic Innervation

Besides Purkinje dendrites, an equally prominent cellular component of the cerebellar cortex are the BG fibers [15] (Figures 2D–2F and S1). In rodents, BG are present during embryonic stages [23]; they migrate to the cerebellar cortex before birth, and their radial fibers reach the pia to form characteristic endfeet by late embryonic stages [14]. BG fibers thus represent the earliest radial structures across the cerebellar cortex, before the arrival of Purkinje neurons [14]. During the first postnatal week, BG fibers are thin, smooth, and unbranched. The glia-specific cytoskeleton protein GFAP can be detected by P4 [24]. The simple BG fibers subsequently undergo profound morphological differentiation and maturation [16,18,25]. During the second week, when Purkinje dendrites extend, BG fibers differentiate in a deep to superficial gradient: whereas BG fibers transversing the external granule layer (EGL) remain smooth, they extend coarse lateral appendages in the underlying ML [26]. During the third and fourth week, BG fibers further branch, extend lateral varicoses and fine processes, eventually forming an extensive reticular meshwork [16,18,25]. Consistent with these results, using single-cell electroporation to label BG with GFP, we found that BG fibers project highly irregular lateral branches during the third postnatal week (Figure S4A). Furthermore, using transgenic mice expressing GFP under the control of a mouse GFAP promoter [27], we were able to visualize the extensive meshwork of the BG system in the ML, and found that GFAP was largely concentrated in the radial BG fibers, but not the finer lateral appendages and processes (Figure S4B). In addition to the apical radial fibers in the ML, BG cell bodies also give off numerous lamellar processes that enwrap Purkinje cell soma and AIS after the third postnatal week [11,14], although the more precise timing of this process is unclear. Mature BG cells are thus highly polarized astrocytes with distinct subcellular specializations.

The vertical bias of the orientation of stellate axon collaterals prompted us to examine their relationship with BG fibers during the development of dendritic innervation. As expected, when stellate cell precursors were migrating across the PCL in the second postnatal week, GFAP-positive BG fibers were prominent throughout the ML (e.g., P8, Figure 2D). Upon reaching their destination in the ML, stellate cells began to extend neurites. Although their axons extended in different directions, many of their descending/ascending branches were strictly associated with GFAP-labeled BG fibers (Figure 2E). This association was particularly prominent in the upper ML, where stellate axons often perfectly followed the curving BG fibers for tens of microns, and remained so in subsequence weeks (Figure 2F). Such extensive association with BG fibers contrasts the rather patchy and “en passant–type” interaction between Purkinje dendrites and BG fibers [14,16,28]. Our detection of association between stellate axons and Bergmann glia was probably an underestimate, since the finer lateral BG appendages were not well labeled by GFAP. Importantly, there was no association between basket axons and BG fibers (Figure 2G), consistent with the finding that basket terminals grow along the proximal dendrite-soma-AIS of Purkinje cells (Figure 1H).

**Figure 3:**
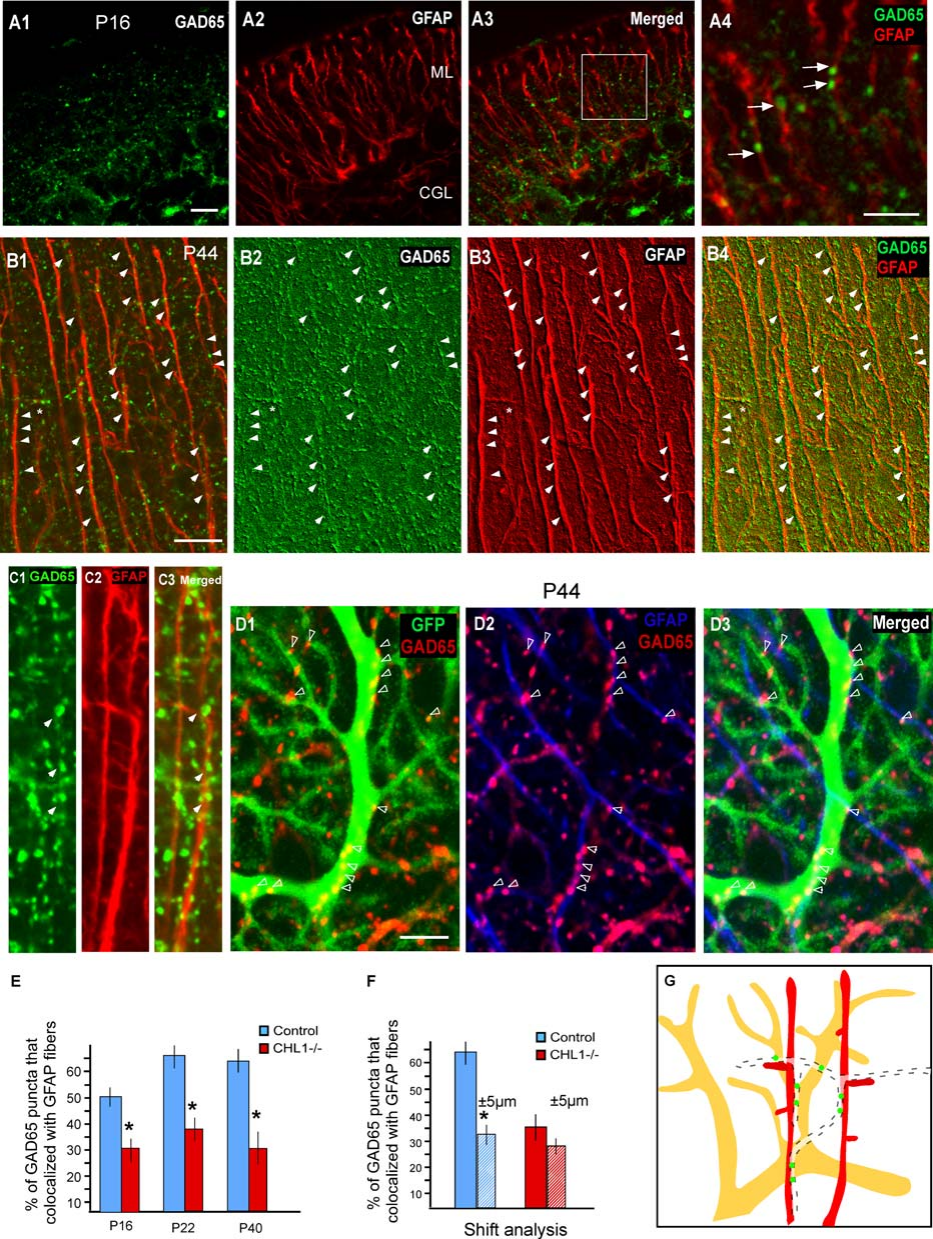
GABAergic Synapses Develop at the Intersection between Purkinje Dendrites and GFAP Fibers (A) At P16, double labeling of GAD65 (A1) and GFAP (A2) revealed a significant colocalization of GABAergic presynaptic boutons and BG fibers in the ML (A3). Note the association of GAD65 with BG fibers in (A4) (arrows), a higher magnification of the boxed area in (A3). (B and C) Prominent colocalization of GAD65 along GFAP fibers at P44. Strings of GAD65 puncta can be discerned that align to the radial GFAP fibers (arrowheads) and also to some lateral appendages (star). (B2–B4) was obtained using the “sharper” filter function of the LSM510 confocal software. (D) Triple labeling of GABAergic boutons (GAD65 in red), BG fibers (GFAP in blue), and a Purkinje dendrite (PV-GFP expression in B20 line; green). GAD65 puncta colocalized with the dendritic shaft of this Purkinje cell (arrowhead in [D1]). Note that the same clusters of GAD65 punta also aligned with GFAP fiber (D2), and GAD65 labeled boutons localized to the intersection between Purkinje dendritic shaft and GFAP positive BG fibers (arrowheads, [D3]). (E) Quantification of GAD65 association with GFAP fibers at P16, P20, and P40 in WT and *CHL1^−/−^* mice. Note that there is a significant reduction of GAD65 association with GFAP at all ages (an asterisk [*] indicates *p* < 0.001). (F) An artificial shift of BG fibers (see Materials and Methods) by 5 μm to the left or to the right induced a 55 ± 7% reduction of their colocalization with GAD65. In contrast, shift analysis performed in image stacks from *CHL1^−/−^* mice did not change the percentage of this association. (An asterisk [*] indicates *p* < 0.001.) (G) A schematic representation of the relationship between stellate axons (dashed lines), BG fibers (red), and Purkinje dendrite (yellow). Stellate axons extend along BG fibers to reach Purkinje dendrite, and synaptic boutons are preferentially formed or stabilized at their interceptions. Scale bars indicate 20 μm.

To substantiate this finding, we further examined the association of GABAergic synaptic markers with BG fibers. GAD65, an isoform of glutamic acid decarboxylase, is localized to GABAergic presynaptic terminals and physically coupled to synaptic vesicles ([29]). The onset of GAD65 expression has been shown to coincide with GABAergic synaptogenesis in the cerebellum [30]. We focused our analysis to the upper ML, where most, if not all, GAD65 signals are derived from stellate cell axons. At P16, shortly after stellate neurons begin to send their axons and made synaptic contacts, double labeling revealed a 52% colocalization between GAD65 and GFAP (Figure 3A). This colocalization increased to 65% in the more mature ML (Figure 3B and 3C). To rule out the possibility that these levels of colocalization can be reached by chance, we artificially shifted the confocal image stacks of GFAP horizontally relative to that of GAD65 by 5 μm, and then reanalyzed GAD65 and GFAP colocalization (since BG fibers were arranged vertically with an average gap of approximately 5 μm between neighboring fibers; see Materials and Methods). This shift analysis revealed a highly significant 30% decrease in GAD65-GFAP association (*p* ≤ 0.01, *n* = 20 different sections in 3 different mice), indicating that the organization of GAD65 along BG fibers was not due to chance. This analysis is likely an underestimate of GAD65–BG association since GFAP antibodies did not label well the finer BG processes (Figure S4B and S4E). In addition to the statistical association, strings of GAD65 puncta, indicative of an underlying stellate axon branch, were frequently seen to perfectly align with GFAP fibers (Figure 3B and 3C). The combined observations suggest that BG fibers in the cerebellar cortex provide a growth template, which may organize stellate axons into characteristic orientations and trajectories. These results are consistent with ultrastructural observations that stellate cell axons and presynaptic terminals are surrounded by glial processes during the third postnatal week [14].

### GABA Synaptic Marker Development at Intersections between Purkinje Dendrites and Bergmann Glial Fibers

Since Purkinje dendrites are the major postsynaptic targets of stellate axons, our results raise the question of whether and how BG fibers guide stellate axons to Purkinje dendrites. Mature Purkinje dendrites bear large numbers of synaptic boutons, but much of their surface is ensheathed by a thin BG process [14]. The glial sheath of a dendritic segment is thought to consist of processes derived from several neighboring BG cells [14]; and it has been recognized since Cajal that the BG fibers are intercalated between the dendritic trees of successive Purkinje cells [11]. Using our Pv-GFP reporter mice, which label individual Purkinje dendrites, and GFAP antibody, we found that BG fibers most often intersected dendritic shafts at sharp angles and did not extend along dendrite at significant length (Figures 3D and S1A). Furthermore, in GFAP-GFP transgenic mice, which occasionally gave sparse labeling of BG cells, double labeling with calbindin antibody showed that a single BG fiber most likely encounters several intercalated Purkinje dendrites (Figure S1B). Therefore, BG fibers impinge upon and enwrap multiple Purkinje dendritic segments in a patchy, en passant pattern.

To examine the precise relationship among stellate cell presynaptic terminals, BG fibers, and Purkinje dendrites, we performed triple labeling with GAD65 and GFAP antibodies in our Pv-GFP mice. As expected, GAD65 puncta colocalized with the shafts of Purkinje dendrites, occasionally aligned in a “beads along a string” pattern, indicative of a stellate axon branch (Figure 3D1). Importantly, the same GAD65 puncta and clusters were also precisely aligned along a GFAP fiber (Figure 3D2), indicating that stellate axon boutons are formed at the intersection between BG fibers and Purkinje dendrites (Figure 3D3). Together, these results suggest that BG fibers in the ML represent an “intermediate scaffold,” which may guide stellate axons to approach Purkinje dendrites in defined orientation and trajectories, and form synaptic contacts at the intersection between BG fibers and Purkinje dendrites (Figure 3G).

### CHL1 Is Expressed along Bergmann Glial Fibers during the Development of Stellate Axon Arbors

**Figure 4:**
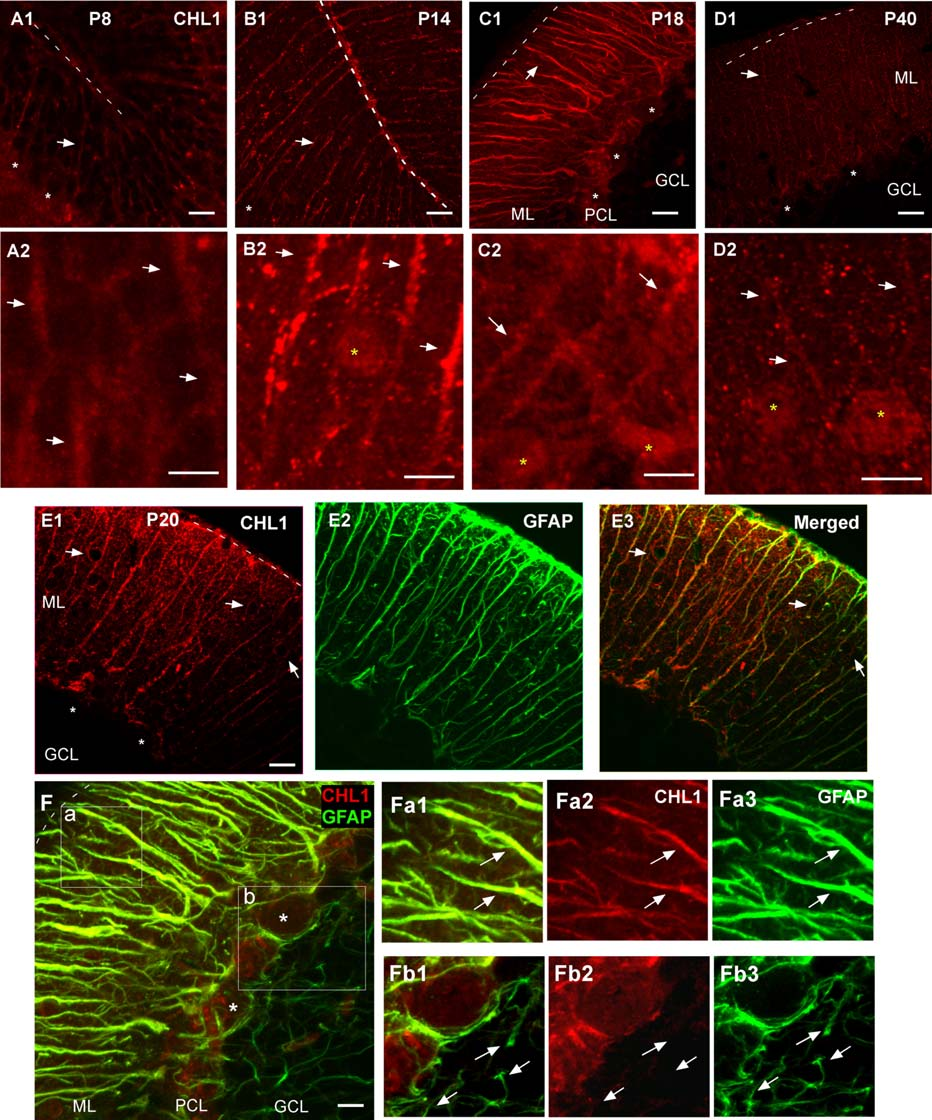
CHL1 Expression in the Developing Cerebellar Cortex (A1–D1) CHL1 was prominently expressed in radial stripe patterns (arrows; corresponding largely to BG fibers) in the ML at P8 (A1) and P14 (B1), reaching higher levels and becoming more diffuse at P18 (C1), and declining in adult (P40, [D1]) mice. Pia is indicated by dotted lines. White stars indicate somata of Purkinje cells. (A2–D2) Images are higher magnification of (A1–D1) and are taken from the upper ML, which contained stellate cell somata. CHL1 expression in stellate cells was undetectable at P8 (A2), appeared around P14 ([B2], yellow star), and remained at P18 (C2) and in adult (D2) mice. (E) CHL1 (E1) closely colocalized with GFAP (E2) in developing cerebellar cortex (E3); only P20 data are shown. Note that CHL1 signals became more diffuse in the ML at this age. (Also see Figure S4.) (F) GFAP (green) labeled both the apical BG fibers in the ML (a) and basal lamellae (b); the latter enwrap Purkinje soma and AIS. At P18, the colocalization of CHL1 and GFAP was restricted to the ML and PCL (inset a, arrows), but not to the basal lamellae in the granule cell layer ([GCL], inset b, arrows). CHL1 (F2a and F2b) is colabeled with GFAP (F3a and F3b). (F1a) and (F1b) are the merged pictures of (F2a and F3a) and (F2b and F3b), respectively Scale bars indicate 20 μm.

To explore the molecular mechanisms underlying the GABAergic innervation of Purkinje dendrites, we took a candidate gene approach and focused on the L1CAMs. The L1CAM subfamily consists of L1, CHL1, NrCAM, and neurofascin [20]. We have previously shown that a Purkinje cell–specific splice variant of neurofascin (NF186) directs the innervation of axon initial segment by basket cell axons [13]. We therefore systematically examined the expression pattern of every L1CAM during the postnatal development of the cerebellar cortex. Interestingly, each member was localized to distinct subcellular compartments in neurons and glia cells (Figure S2). During the third postnatal week (e.g., P16), whereas NF186 was highly restricted to AIS-soma of Purkinje cells [13], L1 was abundantly expressed in parallel fibers and other unmyelinated and premyelinated axons (Figure S2D and S2G). NrCAM was more diffusely (but certainly not ubiquitously) expressed in the ML, although the precise cellular and subcellular locations could not be ascertained (Figure S2E). Interestingly, in the PCL, NrCAM appeared to localize to the basal lamellae of BG that wrapped around Purkinje soma and AIS (Figure S2E). Finally, using an antibody to a peptide epitope in the FNIII domain of CHL1 (Figure 3), we found that CHL1 was distributed in a prominent radial stripe pattern that resembled BG fibers along with diffuse labeling in the ML (Figure 4A–4D). Indeed, CHL1 closely colocalized with GFAP (Figure 4E), but not the Purkinje dendrite marker calbindin (Figure S2F). Such colocalization with GFAP was detected throughout postnatal development (unpublished data).

We further characterized the postnatal developmental expression of CHL1. At P8, when stellate cells were just migrating across the PCL, CHL1 was already prominent along BG fibers (Figure 4A1; colocalization with GFAP not shown). CHL1 was subsequently also detected along the lateral appendages during the second and third week (P14–20, Figure 4C1 and 4E). Importantly, along the polarized BG cells, CHL1 was mainly localized to the apical radial fibers and processes, but not to the basal lamellae that extend towards Purkinje cell AIS (Figure 4F). This pattern in the PCL was clearly distinct from that of NrCAM (Figure S2E). CHL1 expression subsequently diminished in the BG fibers and became more diffuse, yet prominent, in the molecular layer (Figure 4D1). In situ hybridization indicates that CHL1 is also expressed in stellate interneurons and granule cells at P14, but not in mature Purkinje neurons [31]. Consistent with these data, CHL1 immunofluorescence appeared in stellate cell somata as early as P14, and remained at P18 and P40 **(** Figure 4A2–D2). It was difficult to determine whether CHL1 was also distributed along stellate axons and/or dendrites because of the more diffuse labeling in the ML. Lower levels of CHL1 expression in the ML remained in adulthood (in 1-y-old mice, unpublished data).

### Deficient GABAergic Presynaptic Terminals in the ML of *CHL1^−/−^* Mice

**Figure 5:**
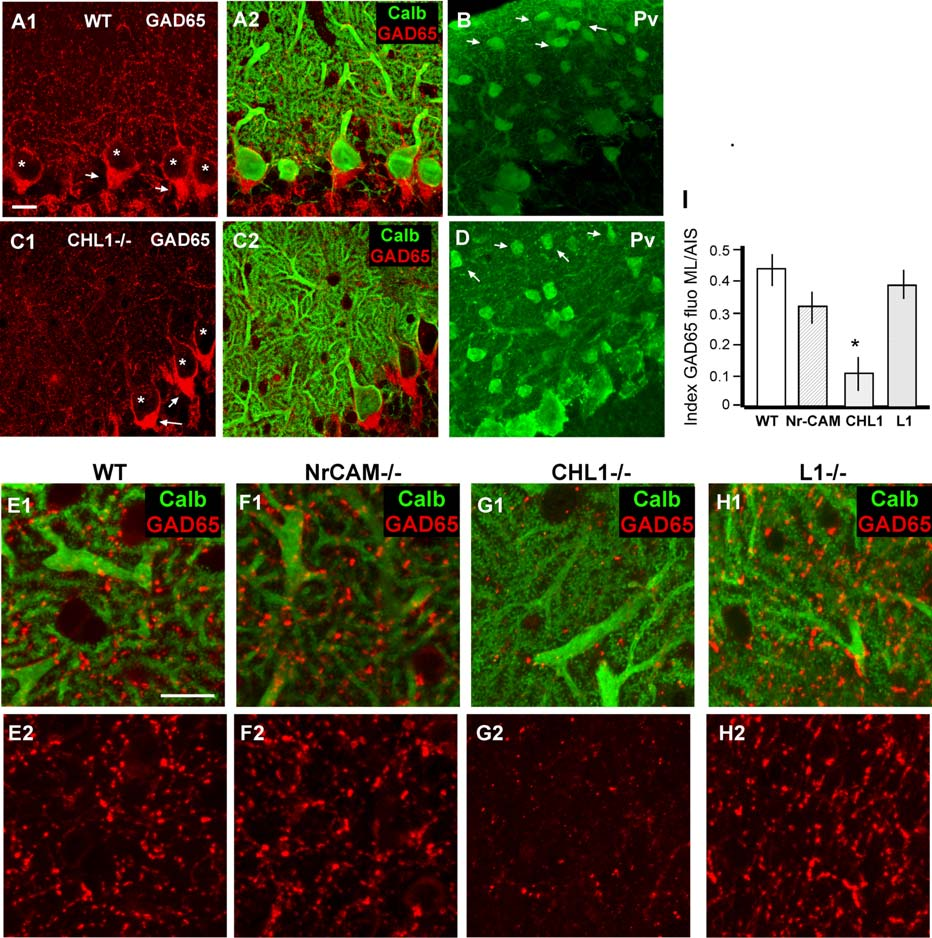
Deficient GABAergic Innervation in the ML of CHL1^−/−^ Mice (A) In WT mice, GAD65 staining revealed GABAergic boutons in the ML and the pinceau synapses at Purkinje cell AISs (arrows). (B–D) In *CHL1^−/−^* mice (C), GAD65 labeling in the ML was significantly decreased, whereas the pinceau synapses remained intact (arrows). Stars indicate Purkinje soma. GABAergic interneurons (arrows in [B] and [D]) labeled with Pv were present in normal density and locations in the ML of *CHL1^−/−^* (D), compared to those of WT (B) mice. (E–I) GAD65 expression in the upper ML of WT (E), *NrCAM^−/−^* (F), *CHL1^−/−^* (G), and *L1^−/−^* (H) mice were quantified as a ratio of fluorescence intensity in ML and at Purkinje AIS. GAD65 (E2, F2, G2, and H2) is colabeled with Calbindin (E1, F1, G1, and H2). (I) There is a 3-fold decreased in this ratio only in *CHL1^−/−^* mice ([I], *p* ≤ 0.001). Purkinje dendrites appeared normal in all phenotypes. Scale bars indicate 20 μm.

To investigate whether CHL1 plays a role in the GABAergic innervation of Purkinje dendrites, we first examined the expression of the presynaptic marker GAD65 in the ML of CHL1 knockout mice (Figure 5A and 5C). As a control, we also surveyed all the viable L1CAM mutant mice using the same assay (Figure 5E–5H). The vast majority of GABAergic terminals in the ML are derived from stellate axons; Purkinje collaterals and basket axons only contribute to a small minority near the PCL [11]. Purkinje dendrites are the predominant targets of stellate axons, although the dendrites of stellate, basket, and Golgi cells are also innervated [11]. In the adult cerebellar cortex (>P40), we found a profound reduction of GAD65 labeling in *CHL1^−/−^* mice, but not in *L1^−/−^* and *NrCAM^−/−^* mice (Figure 5E–5H). This reduction was specific to the ML layer: GAD65 labeling at Purkinje AIS in *CHL1^−/−^* mice was identical to that of wild-type (WT) littermates, *L1^−/−^* mice, and *NrCAM^−/^* ^−^ mice (Figure 5A and 5C, and unpublished data). We took advantage of this result to quantify GAD65 signals in the upper ML as a ratio to those at the Purkinje cell AIS. Such quantification revealed an approximately 60% reduction (*p* ≤ 0.01, *n =* 4 mice) of GAD65 in *CHL1^−/−^* mice compared to their WT littermates (Figure 5I). This significant reduction was not due to a defect in the migration of stellate cells, since stellate cell density and distribution in the ML assayed by Pv immunofluorescence were the same as those in WT mice (Figure 5B and 5D). Furthermore, calbindin staining did not reveal any discernable defects of Purkinje dendrites. We also examined glutamatergic innervation of Purkinje dendrites. The density of parallel fiber synapses and climbing fiber synapses detected by vGluT1 and vGluT2 [32] immunofluorescence, respectively, showed no differences between *CHL1^−/−^* and WT mice (Figure S5A–S5F). The ultrastructures of parallel fiber and climbing synapses also appeared normal (Figure S5G–S5K). These results suggest that, among the L1CAMs, CHL1 appears to play a highly specific role in the GABAergic innervation of Purkinje dendrites. The GAD65 assay itself does not rule out the possibility that stellate innervation of other cell types may also be affected.

Although CHL1 has been shown to modulate radial migration of certain populations of pyramidal neurons in sensory areas of developing neocortex [33], we did not find notable defects in density and position of Purkinje neurons in the cerebellar cortex, although subtle defects cannot be ruled out. We did notice occasional mispositioning of BG cell soma in the granule cell layer (Figure S1C). BG fibers labeled by GFAP were also largely normal, except that they occasionally appeared somewhat less well organized (Figure S1). It is not clear whether these are due to a direct effect of CHL1 deficiency in BG or an indirect consequence of their disrupted association with stellate axons.

### Aberrant Stellate Axon Arborization and Presynaptic Boutons in *CHL1^−/−^* Mice

**Figure 6:**
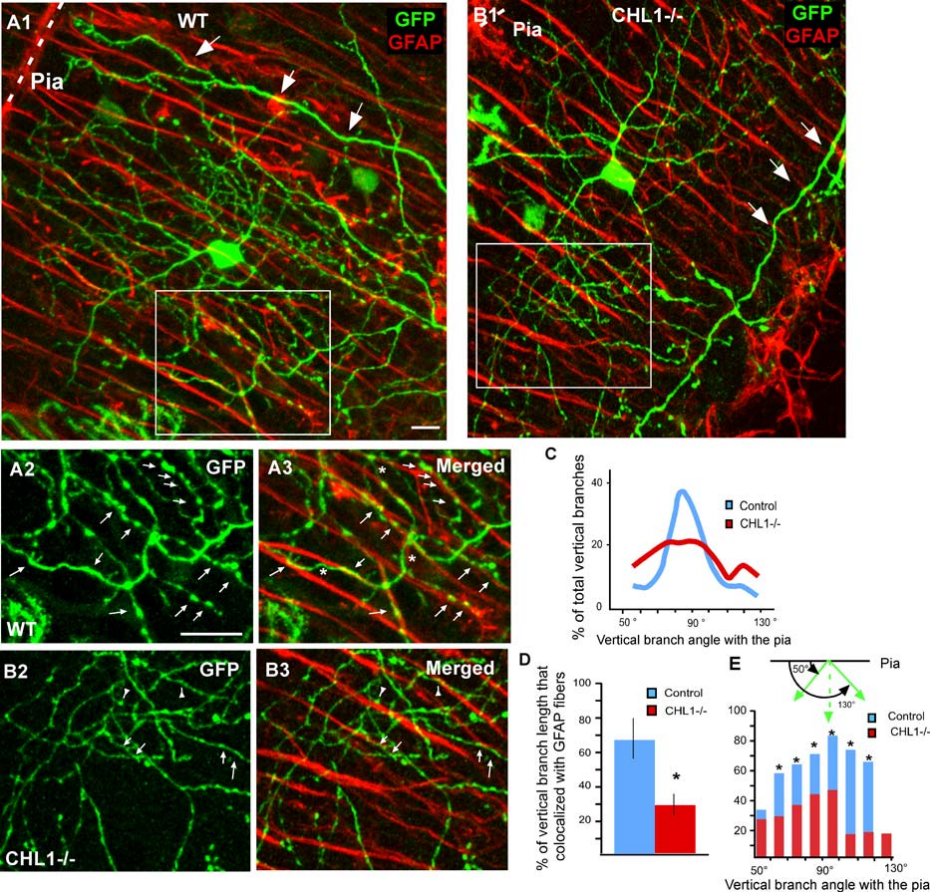
Aberrant Stellate Axonal Arborization in *CHL1^−/−^* Mice (A) (A1) single stellate axon arbor labeled by PV-GFP in the B20 reporter line at P40. Even though the axon arbor was complex, close correlation between axon branches and GFAP fibers can be readily detected ([A2 and A3], arrows). Axon branches often switched between neighboring GFAP fibers (stars). Large arrows in (A1) indicate basket cell dendrites. (A2) and (A3) are higher magnification of the white box in (A1). (B) In *CHL1^−/−^::PV-GFP* mice, stellate axons appeared thinner and disoriented, with much reduced association to GFAP fibers (arrows in [B3]). Axon branches often crossed over BG fibers at sharp angles (arrowheads in [B3]). (B2) and (B3) are higher magnification of the white box in (B1). (C) Quantification of the orientation of stellate axon branches in WT mice revealed a normal distribution towards the pia surface, with 55% of branches oriented at angles between 80°–100° towards the pia. In *CHL1^−/−^* mice, only 32% axon branches were oriented in this range. (D) Quantification of the association of vertically oriented stellate axon branches with GFAP fibers revealed a significant reduction in *CHL1^−/−^* mice (29.6 ± 6.2% ) compared to that in WT mice (69.7 ± 11.1%). (E) Quantification of the association of stellate axon branches oriented at multiple angles with GFAP fibers showed significant reductions in *CHL1^−/−^* compared to WT mice. Note that in the WT, more than 60% of axonal branches colocalized with GFAP, regardless of their angle with the pia (*n* = 400, an asterisk [*] indicates *p* < 0.001 from 10 single cells in each phenotype). Scale bars indicate 20 μm.

**Figure 7:**
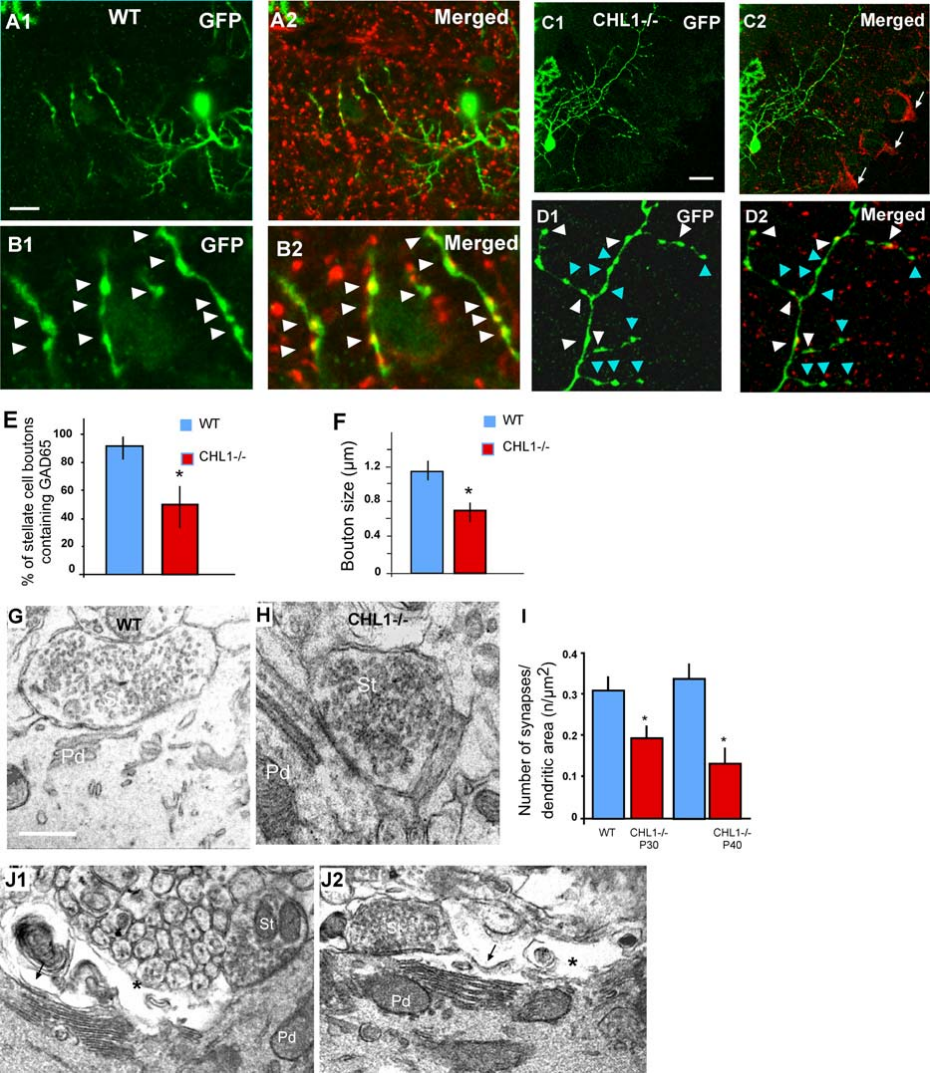
Deficient and Decreased Number of Stellate Synapses in *CHL1^−/−^* Mice. (A) Single stellate cell axons (A1) labeled in WT PV-GFP mice showed large and distinct boutons that colocalized with GAD65 (A2).. (B) Higher magnification view showed that nearly all of these boutons (B1) contained GAD65 ([B2], arrowheads). (C and D) In *CHL1^−/−^::PV-GFP* mice, stellate boutons appeared smaller (white arrowheads), and many of them did not contain detectable levels of GAD65 (blue arrowheads). Note the normal GAD65 signal at pinceau synapses in (C2) (arrows). (E) Quantification of the percentage of stellate boutons colocalization with GAD65 showed a 43% reduction in *CHL1^−/−^* compared to WT mice (*p* ≤ 0.001. (F) Quantification of stellate bouton size revealed a 40% reduction in *CHL1^−/−^* compared to WT mice (*p* ≤ 0.01). (G and H) Ultrastructural analysis showed stellate cell synapses along Purkinje dendritic shafts in the ML of *CHL1^−/−^* mice (H), with largely normal morphology and organelle organization as compared to those in WT mice (G). (I) Quantification of stellate cell synapse density along Purkinje dendritic surface in the upper ML of *CHL1 ^−/−^* mice revealed a approximately 40% reduction compared to WT mice at P30 (*p* < 0.03), and a 60% reduction compared to WT mice at P40 (*p* ≤ 0.001). (J) Ultrastructural analysis showed atrophy of stellate axon terminals in 3-mo-old *CHL1^−/−^* mice. Purkinje dendrites (Pd) were often apposed by degenerating profiles exhibiting electron-dense membrane accumulations (arrows) and electron-lucent empty spaces (stars). (J1) and (J2) are two different examples. Note a symmetrical synapse from most likely a stellate cell axon terminal (St).

To investigate the role of CHL1 in the development of stellate axons, we examined the morphology of single stellate axon arbors using our Pv-GFP (B20) mice. In the ML of mature WT B20 mice (P44), stellate axons display complex arbors with characteristic orientations (Figure 6A); a majority of these axon branches displayed a predominantly vertical orientation and were associated with GFAP-labeled BG fibers (Figure 6C–6E). Quantification of the orientation of stellate axon branches relative to the pia surface revealed that they followed a Gaussian distribution, with a peak between 80° and 100° (Figure 6C). In addition, 70% of these vertically oriented axon branches were associated with GFAP-positive fibers (Figure 6D and 6E). Even when axons branched and turned, they often switched between neighboring BG fibers (Figure 6A3; indicated by arrowheads and stars). Mature stellate axons bore distinct boutons, and more than 90% of these boutons contained the synaptic marker GAD65 (Figure 7A and 7B).

In *PV-GFP(B20)::CHL1^−/−^* littermates, most stellate axons still were able to develop fairly complex arbors at this age, but appeared thinner, more wavy, with significantly altered orientation and trajectories (Figure 6B). When double labeled with GFAP, the notable defects were their reduced association with BG fibers and the reduction of vertically oriented branches. Indeed, the orientation of axon branches was much more evenly distributed (Figure 6C), and many of these more horizontally oriented axons often simply crossed over the BG fibers (Figure 6B3). Quantification revealed that less than 30% of stellate axon branches were associated with GFAP fibers, regardless of their orientation, indicating a significant reduction compared to that in WT mice (Figure 6D and 6E). The altered arbor morphology of stellate axons and their reduced association with GFAP fibers was apparent at P16 and P20 (compare Figure S6A and S6B with Figure 2). In several extreme cases in P44 *CHL1^−/−^* mice, stellate axons were grossly abnormal, with much-reduced branching and simpler arbors. These axons extended rather randomly, twisted, tangled, and even circled around (Figure S6C), with apparently a complete loss of orientation preference. The failure to interact with GFAP fibers may have profoundly altered stellate axonal organization and trajectory in *CHL1^−/−^* mice. These axons also bore smaller boutons (Figure 7C and 7D), and only 50% of these boutons contained detectable GAD65, a 43% reduction compared to WT mice (*p* ≤ 0.001; Figure 7E and 7F). Importantly, these defects were highly specific to stellate axon, basket axons and their innervation of Purkinje AISs appeared entirely normal in *CHL1^−/−^* mice both at single-cell resolution (Figures 7C2 and S7) and when assayed with GAD65 (Figure 5C).

### Deficient Innervation of Purkinje Dendrite by Stellate Axons in *CHL1^−/−^* Mice

We used electron microscopy to directly examine stellate synapses on Purkinje dendrites. We restricted our analysis on the upper third of the ML, where all symmetric synapses are derived from stellate axons. In WT mice at P44, stellate terminals exhibiting symmetric synapses were identified along the Purkinje dendritic shafts as clear varicosities containing densely studded, flattened vesicles (Figure 7G). The density of stellate terminal boutons with symmetric synapses was quantified against Purkinje dendritic surface area from serial ultrathin sections to avoid overlooking stellate terminal profiles. In *CHL1^−/−^* littermates, morphologically normal terminal boutons with symmetric synapses were clearly present along Purkinje dendrites (Figure 7H), with diameters ranging from 0.4–0.7 μm, and an active zone length of 0.15–0.26 μm. However, the density of symmetric synapses was reduced by 60% (*p* ≤ 0.001). At P30, there was also a significant reduction in the density of symmetric synapses by approximately 40% (*p* < 0.03). On the other hand, basket axon synapses on Purkinje somata, parallel fiber synapses on dendritic spines, and climbing fiber synapses on dendritic shafts were all indistinguishable between P44 WT and *CHL1^−/−^* mice (Figures S5G–S5K, S7C, and S7D).

Much more severe defects of stellate axon terminals in *CHL1^−/−^* mice were detected at older ages. In 3-mo-old mutants, degenerating axon profiles were frequently seen in the upper ML, exhibiting electron-dense membrane accumulations and electron-lucent empty spaces (Figure 7J). On the other hand, nearby climbing fiber terminal profiles along the same Purkinje dendrites were perfectly normal. Together, these ultrastructural results suggest that in the absence of CHL1, aberrantly organized and oriented stellate axons can still manage to contact Purkinje dendrites and form synapses, but at significantly reduced efficiency and density. In addition, these synapses cannot be maintained, leading to atrophy of stellate axon terminals.

### CHL1 in Bergmann Glia Contributes to Stellate Innervation of Purkinje Dendrites

**Figure 8:**
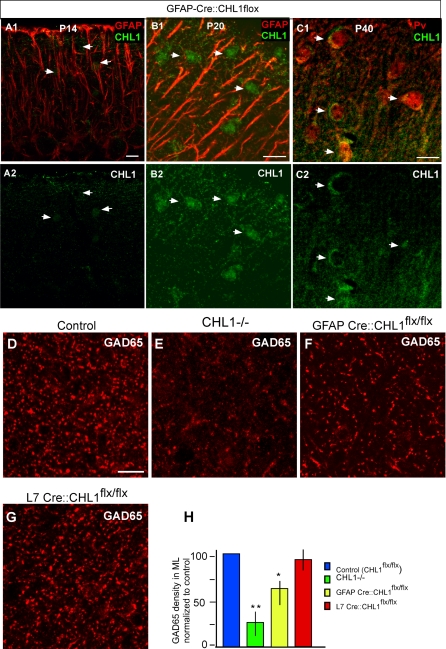
Conditional Deletion of CHL1 in Bergmann Glial Cells Results in Significant Reduction of GAD65 Expression in the Upper ML (A–C) BG-restricted CHL1 deletion was achieved by breeding CHL1^flx/flx^ and GFAP-Cre mice. At both P14 (A) and P20 (B), CHL1 (A2 and B2) was no longer present as stripe patterns that colocalize with GFAP (red in [A1] and [B1]) in the ML as in WT mice (Figure 4); but CHL1 was detected as somata profiles (arrows) and diffuse signals in the ML, especially at P20 and P40 (C2). (C1) showed CHL1 colabeled with calbindin (red). (D–G) CHL1 expression remained at low levels in the ML at P40 both as diffuse signals and also outlined stellate cell somata, which were positive for Pv ([D], Pv in red). GAD65 expression in the upper ML of CHL1^flx/flx^ control (D), germline *CHL1^−/−^* (E), GFAP-Cre::CHL1^flx/flx^ (F), and L7-Cre::CHL1^flx/flx^ (G) mice. (H) Compared to GAD65 labeling in the ML in control mice, mice lacking CHL1 in BG showed a 27% reduction (an asterisk [*] indicates *p* ≤ 0.01, *n* = 4), germline *CHL1^−/−^* mice showed a 65% reduction (double asterisks [**] indicate *p* ≤ 0.001, *n* = 4), and L7-Cre::CHL1^flx/flx^ mice showed no reduction. Scale bars indicate 20 μm.

Besides BG, CHL1 is also expressed in other cell types, such as stellate cells, granule cells, and their parallel fibers in the developing cerebellum [31]. To further investigate the role of CHL1 in BG, we bred a conditional CHL1 mutant strain (CHL1^flx^) (see Materials and Methods) with a transgenic lines expressing CRE recombinase under the control of GFAP [34]. At P14, P20, and P40 in GFAP-Cre::CHL1^flx/flx^ mice, CHL1 expression was undectable along BG fibers but was clearly present in stellate cells (Figure 8A–8C). At P40, there was a significant reduction of GAD65 density in GFAP-Cre::CHL1^flx/flx^ mice compared to CHL1^flx/flx^ controls (Figure 8D, 8F, and 8H; 27 ± 7%; *p* ≤ 0.05). We also deleted CHL1 in Purkinje cells by breeding CHL1^flx^ mice with the L7-Cre transgenic mice [35]; there was no reduction of GAD65 density at P40 (Figure 8G and 8H), suggesting that CHL1 in Purkinje cells, if any, was not involved in the development of stellate synapses. These results suggest that CHL1 expression in BG contributes to the development of stellate cell synaptic innervation in the ML. Compared with germline *CHL1^−/−^* mice (Figure 5E and 5H), the intermediate reduction of GAD65 in the ML of GFAP-Cre::CHL1^flx/flx^ may be due to two reasons. First, the association between stellate axon and BG fibers may be mediated by CHL1 homophilic as well as heterophilic interactions; absence of CHL1 in BG fibers thus partially impairs the association of stellate axons with BG fibers and the innervation of Purkinje dendrites. CHL1 expression in stellate cells likely also plays a significant role. Second, it is possible that only the GABAergic innervation of Purkinje dendrites (but not stellate dendrites, for example) is guided by CHL1 expression on BG fibers; absence of CHL1 in BG fibers thus only partially reduced the GAD65 signal in the ML.

## Discussion

**Figure 9:**
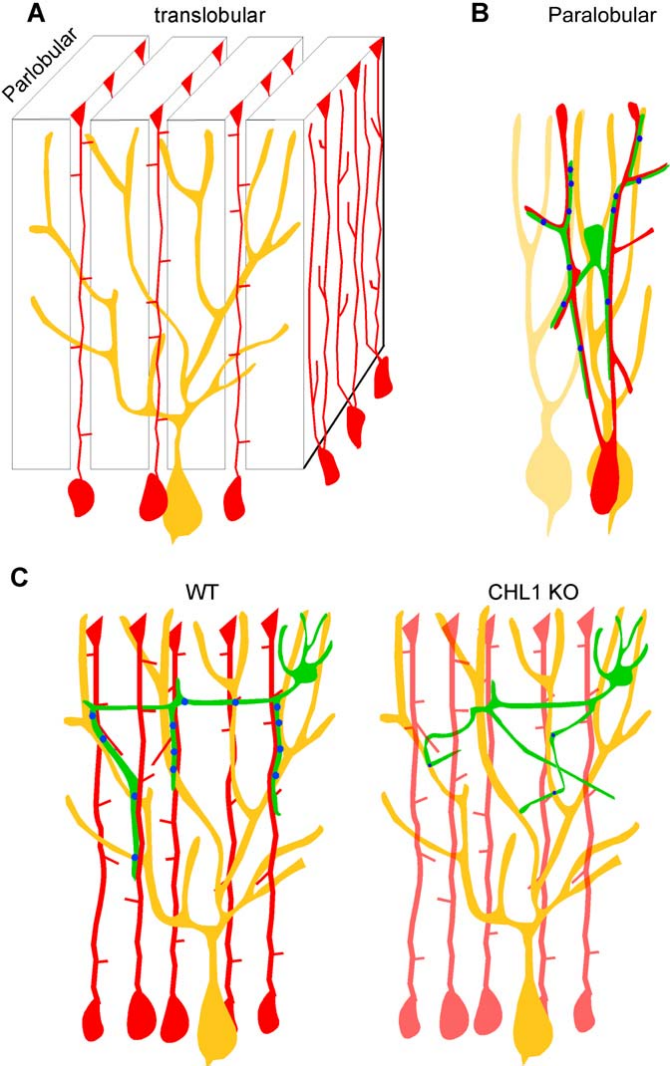
Schematic Representation of the Bergmann Glial Fiber Scaffold and CHL1 in Directing Stellate Axons to Innervate Purkinje Dendrites (A) In the cerebellar cortex, Purkinje dendrites (yellow) are restricted in the translobular plane. Each BG cell (red) gives rise to several ascending BG fibers, which extend in both the translobular and parlobular plane [14,15]. These largely radial fibers from neighboring BG cells further aligned into thin walls, or palisades, in the parlobular plane, perpendicular to the Purkinje dendrites. As a consequence, several BG palisades cut across and impinge upon an individual Purkinje dendrite in a largely vertical orientation. (B) A stellate axon likely contacts segments of multiple intercalated Purkinje dendrites (parlobular view; neighboring Purkinje dendrites are represented in different shades of yellow). Stellate synapses (blue dots) are formed or stabilized at the interception of BG fibers and Purkinje dendrites. (C) In the translobular plane in WT mice (left panel), stellate axons (green) associate with and extend along BG fibers, and are thus organized into characteristic orientations and trajectories towards Purkinje dendrites. In CHL1-deficient mice (right panel), stellate axons can no longer associate with BG fibers, show aberrant orientation and trajectory, and are deficient in synapse formation and/or stability.

The spatial distribution of a neuron's output is determined by the geometry of its axon arbor and the pattern of its innervation. Different classes of neurons often display characteristic axon arbors which target restricted spatial locations, cell types, and subcellular compartments in neural circuits. Some of the best examples of neuronal class-specific innervation patterns are found along Purkinje neurons, which reside in the translobular plane of the cerebellar cortex and receive four sets of synaptic inputs (Figure 1A) [11]. Among the glutamatergic inputs, the parallel fibers run along the parlobular axis and impinge upon Purkinje dendrites perpendicularly; each granule cell axon often contacts a single spine from one entire Purkinje dendrite, but may innervate hundreds of dendrites along its path. In sharp contrast, the climbing fibers restrict their arbors in the translobular plane; each eventually innervates only one Purkinje cell but with hundreds of synapses along its dendritic shaft. The two types of GABAergic interneurons both extend their axons within a rather narrow translobular plane and innervate multiple targets within a few rows of Purkinje cells [11,14]. Whereas a basket axon typically grows along and innervates seven to ten Purkinje somata and AISs [11], the descending and ascending collaterals of a stellate axon likely innervate multiple Purkinje dendrites [11] but do not grow along Purkinje cells (Figure 1). The geometric and subcellular organization of dendritic-targeting GABAergic axons and innervation patterns are crucial in the physiological control of synaptic integration in postsynaptic neurons, yet the underlying mechanisms are largely unknown. Here, we present evidence that stellate axons are organized in characteristic trajectories and guided to Purkinje dendrites by an intermediate scaffold—the BG fibers; in addition, the L1CAM CHL1 is a molecular signal that contributes to the patterning and subcellular organization of stellate axons and innervation (Figure 9).

### Bergmann Glial Fibers as an Intermediate Scaffold for the Patterning and Subcellular Guidance of Stellate Cell Axons

In mature cerebellar cortex, each BG cell gives rise to several ascending BG fibers, which extend approximately 40–50 μm in the translobular plane and 15–20 μm in the parlobular plane [14,15]. Interestingly, these largely radial fibers from neighboring BG cells are further aligned as thin walls, or palisades, in the parlobular plane perpendicular to a Purkinje dendrite, which extends approximately 300–400 μm in the translobular plane and 15–20 μm in the longitudinal plane [14,15]. The consequence of these arrangements is that several BG palisades cut across a single Purkinje dendrite [14,15]. Although this striking spatial organization of BG fibers has long been recognized and postulated to contribute to the architecture of the cerebellum, no specific neuronal elements and developmental process have been identified that rely on such fine arrangement. By high-resolution labeling of stellate axons superimposed upon BG and Purkinje cells, we realized that BG fibers may be an ideal intermediate scaffold to “presort” a stellate axon into characteristic trajectories and distribute them towards multiple Purkinje dendrites.

During cerebellar development, BG fibers represent the earliest radial structures across the cerebellar cortex, even before the arrival of Purkinje neurons [14,23]. The initially simple BG fibers undergo dramatic differentiation and maturation in the second to fourth postnatal week and are transformed into a highly elaborate meshwork, dominated by the vertical palisades [14,16,18,25,26]. Although the elaborate BG fibers appear to be positioned to interact with multiple neuronal components, such as migrating granule cells [19,36], and likely contribute to multiple aspects of cerebellar circuit assembly at different developmental stages, our discovery of their close association with the developing stellate axons is particularly compelling. First, the association was apparent as soon as stellate cells begin to extend axons during the second postnatal week. Second, stellate axons often strictly followed the curving contours of BG fibers for tens of microns, as well as the lateral appendages of BG fiber. Finally, the association between BG fibers and stellate axons was specifically disrupted by the loss of an immunoglobulin family cell adhesion molecule expressed in both BG fibers and stellate cells. It is thus likely that BG fibers mainly serve as a growth template for stellate axons, and additional molecular and/or activity-dependent mechanisms may regulate the size and exuberance of axon arbors. Interestingly, BG processes also express GABA_A_ receptors that enwrap inhibitory synapses [37]; it is thus possible that BG fibers may respond to GABA signaling from developing stellate cell axons. Mature stellate axons extend characteristic ascending and descending collaterals as well as plexus of finer branches and terminals [11]. Our GFAP labeling of BG likely underestimated their association with stellate axons. It is possible that the GFAP-positive BG fibers may represent “highways” for stellate axon collaterals, and that the lateral appendages and processes may serve as “local roads” for axon terminals to approach and innervate Purkinje dendrites.

In both invertebrates and vertebrates, the crucial role of glia cells in axon guidance has been well recognized [38,39]. Glial cells can function as guideposts to attract [40–42], repel [43–45], or stop [46] growth cones of projection neurons [38,47], and can also serve as intermediate targets to coordinate pre- and postsynaptic interactions [46,48]. In the developing rodent olfactory bulb, radial glial cells interact with olfactory receptor neuron axons [49] and have been postulated to contribute to the formation of glomeruli [50]. At hippocampal excitatory synapses, astrocytes form tripartite complexes with pre- and postsynaptic structures, and regulate synapse morphogenesis and maturation [51,52]. Here, we provide the first evidence to our knowledge that the characteristic astroglial processes organize the axon trajectory of GABAergic interneurons and contribute to the establishment of precise patterns of connectivity in complex local circuits, including subcellular synapse targeting. In many areas of the vertebrate brain (e.g., neocortex and hippocampus), highly abundant and morphologically elaborate astrocytes mature during postnatal development along with the assembly of local circuits. It is thus possible that an astroglial intermediate scaffold might be a more general mechanism for directing the trajectory of axon extension, pre- and postsynaptic target interaction, and complex patterns of innervation.

### CHL1 as a Molecular Signal for the Patterning and Subcellular Organization of Stellate Axons

Like other members of the L1CAM [20], CHL1 is expressed in both neurons and glia [31,53]. Although there is evidence that CHL1 promotes and inhibits neurite outgrowth in vitro through both heterophilic and homophilic interactions, respectively [54,55], and may regulate hippocampal axon projection and organization [56,57], the cellular interactions involved and the logic of CHL1′s action have been unclear. The well-defined architecture and connectivity in cerebellar cortex present an advantage in defining the cellular and subcellular expression of CHL1 and in dissecting its role in axonal and synapse development. CHL1 is prominently localized to apical BG fibers since the first postnatal week, and subsequently extend to the lateral appendages during the second and third postnatal week. We cannot ascertain whether CHL1 is also expressed in the fine BG fiber processes (due to the presence of CHL1 in parallel fibers and possibly other neural elements in the ML). Importantly, CHL1 is not localized to the basal lamellae of BG cells, which extend to Purkinje soma and AIS. Such polarized distribution of CHL1 in BG cells may present a permissive substrate for the growth and patterning of stellate axons and for their restriction to the ML to innervate Purkinje dendrites. In addition, CHL1 is expressed in stellate cells, but not in Purkinje neurons [31]. CHL1 immunoreactivity could be clearly detected in stellate cell somata by P14 (Figure 4), although its subcellular distribution (on axons and dendrites) was difficult to discern.

In our analysis of *CHL1^−/−^* mice, the trajectory and orientation of stellate axons and their innervation of Purkinje dendrites were profoundly aberrant. In contrast, basket axons and their innervation of the Purkinje soma-AIS were entirely normal. In addition, Purkinje dendrites and their glutamatergic innervation by climbing fibers and parallel fibers also appeared intact, even though CHL1 is known to be expressed in granule cells and parallel fibers [31,53]. These results reveal a highly specific role for CHL1 in the patterning of stellate cell axon arbors. The significant reduction of GAD65 puncta in the ML may result from a reduction in the number of stellate synapses, deficient synapses, or both. Whereas double labeling and confocal microscopy detected a reduced localization of GAD65 to stellate boutons, ultrastructural analysis confirmed a significant reduction of stellate synapses along Purkinje dendrites. Interestingly, a recent study shows that CHL1 is localized at presynaptic terminals of glutamatergic and GABAergic axons in dissociated hippocampal cultures [58]; CHL1 appears to be targeted to synaptic vesicles by endocytosis in response to synapse activation and regulates the uncoating of clathrin-coated synaptic vesicles [58]. It is thus conceivable that the absence of CHL1 in stellate cell axons may impair GABAergic vesicle endocytosis and GAD65 synaptic localization. We suggest that CHL1 deficiency results in dissociation of stellate axons from their normal BG fiber “tracks,” aberrant axon orientation and trajectory, which contribute to subsequent deficiency in synapse formation and stability. Our current analysis cannot distinguish whether the decreased number of GABAergic synapses from stellate onto Purkinje cells in *CHL1^−/−^* mice results from the inability of the stellate axon to engage in synapse assembly, deficient cell adhesion prior to synapse assembly, or deficient synapse maintenance.

The reduction of GAD65 signals in the ML of BG-restricted CHL1 knockouts further pinpoints a specific role of CHL1 in BG fibers. On the other hand, the intermediate phenotype in these mice compared to that in germline *CHL1^−/−^* mice implies that CHL1 in other cell types, e.g., stellate and granule cells, may also contribute to their axon and synapse development. It is possible that CHL1 may localize to stellate axons and contribute to arbor patterning through homophilic interaction with CHL1 distributed on BG fibers. On the other hand, unknown CHL1 ligands in stellate cells and BG fibers may mediate heterophilic interactions during stellate axon development. Indeed, CHL1 can act as a coreceptor for neuropilin-1 to mediate axon guidance by semaphorin3A during development of the thalamocortical projection [59]. During the third postnatal week, CHL1 expression in stellate cells might also promote the maturation and stability of synaptic innervation through heterophilic interactions with Purkinje dendrites and hetero- or homophilic interaction with BG fibers. Finally, CHL1 might also be localized to stellate dendrites, which are innervated by other stellate axons. Deficiencies in stellate axon arbor and synaptic innervation in *CHL1^−/−^* mice may contribute to the impairment in their motor behaviors, such as the ability to maintain balance on an accelerated Rota-Rod [60].

### L1CAMs and the Subcellular Organization of GABAergic Axons along Purkinje Neurons

Although it was once debated whether basket cells and stellate cells were variants of the same class of cerebellar interneurons, it is now established that they constitute distinct cell types, likely with distinct genetic origins [61], and a fundamental difference is their subcellular target innervation. In both cell types, the final axon arbor and innervation pattern is achieved through sequential developmental processes, which may involve: the pattern and order of their migration into the ML, the elaboration of axon arbors along defined cellular substrates and adhesion mechanisms, and the formation–stabilization of synaptic contacts along different compartments of Purkinje neurons. Here, we demonstrate that stellate and basket cells deploy different cellular and molecular mechanisms to achieve their distinct axon arborization and innervation patterns.

The basket cells make synaptic contacts along the soma-AIS of a Purkinje cell, a highly restricted synaptic target area. It is perhaps not surprising that basket axons arrive at their destination, in part, by growing along the Purkinje proximal dendrite-soma-AIS, guided by a subcellular gradient of neurofascin [13]. The stellate cells, on the other hand, face a rather different task when innervating Purkinje dendrites: even though each Purkinje dendrite is a largely 2-dimensional, flat target, it expands hundreds of microns in the translobular plane. Unlike a climbing fiber, which adheres to and monopolizes a single Purkinje dendrite, a stellate axon innervates segments of multiple dendrites, often with characteristic descending and ascending collaterals. It is thus not obvious how stellate axons can ever achieve such a distinct innervation pattern by direct and strong adhesion to Purkinje dendrites. The BG fibers seem to provide a useful solution to this problem. As an extensive and largely radial scaffold in the ML, the BG fibers are well suited to organize and deliver stellate axons to Purkinje dendrites, with defined orientations and trajectories. In addition, by relying on a glial instead of neuronal substrate, stellate axons may reduce the risk of making ectopic and unnecessary synaptic contacts. Furthermore, the BG fibers appear to direct both the patterning of axon arbor and subcellular innervation. It remains to be investigated whether such an intermediate glial scaffold is a more general strategy to sculpt precise neuronal connections in other brain areas.

We present evidence that, CHL1, a close homolog of neurofascin186, is involved in the development of stellate axons and their dendritic innervation. Our results suggest that different members of the L1 family may contribute to axon patterning and subcellular synapse organization in different cell types, and may act in glia as well as in neurons. The subcellular recruitment of NF186 is achieved by the ankyrinG membrane adaptor protein at the Purkinje AIS [13]. It is tempting to speculate that another form of ankyrin in BG cells may organize CHL1 subcellular localization. In addition to permissive/attractive signals, such as NF186 to basket axons and CHL1 to stellate axons, repulsive or bifunctional signals (depending on different axon types) at distinct subcellular sites may also contribute to topographically precise synapse organization. The identification of physiological ligands for NF186 and CHL1 in basket and stellate axons will further our understanding of the underlying molecular mechanisms.

## Materials and Methods

### BAC transgenic mice.

BAC clones containing the mouse *parvalbumin* (*PV*) genes were identified from the RPCI-23 library (CHORI). A BAC clone containing the entire *PV* gene and approximately 150 kb of upstream and 25 kb downstream regions was used for BAC modifications. A GFP expression cassette was inserted in the first coding exon at the translation initiation site using a procedure developed by Yang et al. [62]. Circular BAC DNAs were injected into the fertilized eggs of the C57BL/6 strain at a concentration of 0.5 ng/μl in microinjection buffer (10 mM Tris [pH 7.4], 0.15 mM EDTA [pH 8.0]) using standard procedures and as described previously (Ango et al., 2004 [13]). Five transgenic founders were identified by PCR and confirmed by southern blotting. All founder lines resulted in germline transmission. GFP expression was first analyzed in fixed brain sections immunolabeled with antibodies to various GABAergic interneuron markers: Pv, somatostatin, calretinin, and VIP. In the cerebellum, different fractions of Purkinje, basket, and stellate neurons expressed GFP among different transgenic lines, from a few percent (the B20 line) to near 100% (the B13 line, and unpublished data), likely due to different genomic integration sites of the transgene. The onset of GFP usually started in the late second postnatal week and increased to higher levels by the fourth week. The GAD67-GFP reporter mice were described in Ango et al., 2004 [13].

### Mutant mice.

The *CHL1^−/−^* mice were described in [56]. *L1^−/−^* and *NrCAM^−/−^* mice were provided by Drs. Dan Felsonfeld and Dr. Martin Grumet, respectively. The CHL1 conditional mutant will be published elsewhere (Kolata et al., unpublished data). The L7-cre mice [34] were obtained from Mutant Mouse Regional Resource Centers (MMRRC) and the GFAP-cre mice [33] from JAX Mice.

### Immunohistochemistry and confocal microscopy.

Mice were anesthetized (sodium pentobarbitone, 6 mg/100 g of body weight) and transcardially perfused with 4% paraformaldehyde in phosphate buffer (pH 7.4). Sagittal sections (80-μm thick) were cut from the cerebellum using a vibratome (Leica VT100). Brain sections were blocked in 5% NGS and 0.1% Triton X-100, and immunostained with antibodies against GAD65 (monoclonal antibody, 1:1,000; Boehringer), GFP (rabbit or chicken polyclonal antibody, 1:500; Chemicon), Pv (monoclonal antibody, 1:1,000; Sigma), CHL1 (chicken polyclonal antibody, 1:500), calbindin (rabbit polyclonal antibody, 1:1,000; Swant), and GFAP (rabbit polyclonal; Geko). Sections were incubated with either Alexa594-conjugated goat anti-mouse or anti-rabbit IgG and Alexa488-conjugated goat anti-rabbit or anti-chicken IgG (1:500; Molecular Probes) and mounted. Sections were imaged using a 63× water immersion objective (Zeiss) using a confocal microscope (Zeiss LSM510) under the same conditions. Scans from each channel were collected in multiple-tracks mode and subsequently merged. Care was taken to use the lowest laser power, and no bleedthrough was visible between the Alexa594 and Alexa488 channels.

### Analysis of stellate axons.

*SHIFT analysis.* All confocal images were acquired using the same microscope setting. Confocal stacks were first merged using maximum transparency setting. The maximum *Z* stack used was 2 μm. Using the ImageJ software, the green (GFAP) and the red (GAD65) channels were then separated and transformed into grey-level 8-bit images before being thresholded. The minimum size of GAD65 puncta was set to between 12 to 750 pixels (signals smaller or larger would not count it). The total number of GAD65 puncta (*X*) was measured using the dot counting function of ImageJ. The grey color images of GFAP and GAD65 were then remerged. Since both images were grey, those GAD65 puncta that colocalized with GFAP were fused into the GFAP signals (as “bubbles along fiber”-like patterns) and would be excluded from the counting procedure set above. Thus in the remerged image, only the GAD65 puncta that were not colocalized with GFAP (*Y*) were counted. With this approach, we were able to count the number of GAD65 puncta in a nonbiased way using the counting function of ImageJ. We then obtained the percentage of GAD65 puncta that colocalized with GFAP as (*X* − *Y*) / *X* * 100. The same procedure was used before and after shifting the GFAP image relative to that of GAD65 by ±5 μm. The Wilcoxon signed rank test was used for paired comparisons of GAD65 puncta density and colocalization with GFAP. Significance was set at *p* < 0.05, and values are means ± standard deviation (s.d.)

*Analysis of stellate axon orientation and their association with GFAP*. All analyses were done blind to the genotype. All ascending and descending axonal branches with a length greater that 4 μm was included in our analysis. Axonal branches of individual neurons were visualized with GFP in our PV-GFP (B20) mice (eight neurons in at least five different mice in each genotype). From each selected axonal branch, its length (*X*) was first measured with the LSM confocal software (Zeiss). The axonal length that colocalized with GFAP (*Y*) was then measured; and the proportion of the branch that colocolized with GFAP was obtained as *Y* / *X* * 100. We measured the angle of each axon branch in relation to the pia surface, which was defined as a horizontal line in our projected image at the 0° angle. An axon branch that was perfectly perpendicular (ascending or descending axonal branches) to the pia would be at the 90° angle. We set a virtual horizontal line (the closest to the pia surface orientation) for each branch and measured its angle in relation to the virtual horizontal line. We took into account only branches with angles between 50° to 130°; and these angles of axon branches were grouped into 10° bins. Values in each bin were pooled together and analyzed with Kaleidagraph (Synergy Software) or Excel (Microsoft) software.

### Statistics.

Paired comparisons of GAD65 density and colocalization with GFAP used the Wilcoxon signed rank test. Significance was set at *p* < 0.05. Parameter values are means ± s.d.

### Antibody production.

Antibodies against CHL1 (CS1123) were raised in chicken against a peptide with the sequence SLLDGRTHPKEVNILR corresponding to the region within the third FNIII domain of the protein plus an N-terminal cysteine for coupling. The specificity of the CHL1 antibodies was confirmed using *CHL1^−/−^* mice, and COS cells transfected a CHL1 expression construct (Figure S2). Production and IGY purification was done by Covance Immunology Service. Similar staining patterns, but higher intensity, were seen with a polyclonal antibody from R&D Systems.

### Electron microscopy.

Brains were perfusion fixed according to routine procedures as described earlier [63]. Briefly, deeply anaesthetized mice were transcardially perfused with a brief rinse in phosphate buffer, 0.1 M (pH 7.4), followed by a solution of 4% freshly depolymerized paraformaldehyde and 0.1% glutaraldehyde in phosphate buffer, supplemented with 2% PVP and 0.4% NaNO_2_. The brains were removed from the skull and left in the same fixative for at least several days. Sagittal vibratome sections of 50–60 μm were postfixed in 1% osmium tetroxide with 1% sodium ferricyanide in 0.1 M cacodylate buffer for 20 min, dehydrated in series of ethanol, and then flat embedded in epoxy resin. Semithin sections (1 μm) were cut and stained with toluidine blue and used for orientation purposes. Ultrathin sections of selected areas of the cerebellar cortex with reference to the ML were cut, using an ultratome LKB IV (Reichert-Jung). and collected on single-slot grids or 75-mesh grids coated with Formvar (Electron Microscopy Sciences) Ultrathin sections were contrasted with uranyl acetate and lead citrate, and analyzed in a Philips CM 100 transmission electron microscope (FEI Electron Optics).

Morphological analysis. Synapses were defined by the presence of a clear postsynaptic density facing a number of synaptic vesicles. By means of a goniometer, sections could be tilted in the beam, thereby determining the symmetry or asymmetry of the synaptic profiles. Measurements of profile length and diameter were made using a morphometric program (Soft imaging system SIS; Olympus).

### In vivo electroporation.

P3–P5 pups were anesthetized with ketamine (0.56 mg/g; xylazine, 0.03 mg/gm body weight). After incision of the skin overlying the skull, a small hole was made directly over the left hemisphere of the cerebellum. A patch pipette filled with 1–2 μl of GFP DNA construct (endotoxin-free preparation) were injected directly into the tissue (1 μg/μl DNA), and mouse pups were subjected to electric pulses (four to six pulses at 200 mv/cm for 50 ms with intervals of 950 ms) by gold-plated electrode (BTX) placed directly on the skull. The skin was then sutured. After recovery from anesthesia, pups were returned to mother under standard housing. Mice were then sacrifice at P16 and analyzed.

## Supporting Information

Figure S1 Relationship between Bergman Glia and Purkinje Dendrites (A) A single Purkinje neuron from a PV-GFP (B20) mouse colabeled with GFAP immunofluorescence (red) revealed patchy, en passant–type, rather than extensive, association between BG fibers and Purkinje dendrite (arrowheads).
(B) Complete BG fibers visualized by GFAP-GFP mice colabeled with calbindin (red) showed that a single branch of BG fiber likely impinges upon multiple intercalated Purkinje dendrites in a patchy manner (arrowheads).
(C and D) In *CHL1^−/−^* mice, the GFAP fibers, BG vertical palisades (arrowheads), and lateral appendages (stars) of BG fibers all appeared similar to those in WT mice; there was occasional mispositioning of BG cell soma (C2, arrows). Scale bars indicate 20 μm.
(8.72 MB TIF)

Figure S2 L1CAM Expression Pattern in Cerebellar Cortex at P16 (A–D) Members of L1CAMs were differentially localized to subcellular compartments in neurons and glia cells in cerebellum at P16. (A) Neurofascin186 was highly restricted to AIS-soma of Purkinje cells. (B) NrCAM was more diffusely, but not ubiquitously, expressed in the ML. (C) CHL1 was distributed in a prominent radial stripe pattern. (D) L1 was abundantly expressed in parallel fibers and other unmyelinated and premyelinated axons. Purkinje cells were labeled by either Pv (B2 and D2) or calbindin (A2 and C2) antibodies.
(E) A high-magnification view of NrCAM colabeled with GAD65 in the ML, PCL, and granule cell layer (arrows). Note that NrCAM enwrapped GAD65-positive pinceau synapses at Purkinje AIS (arrow), suggesting its localization to the basal lamellae of BG cells.
(F) No coalignment of stripe patterns of CHL1 immunofluorescence (red) with Purkinje dendrite (calbindin, green).
(G) L1 is prominently expressed by granule cell axons and likely other unmyelinated axons. Note the fiber-like labeling in the molecular layer (G1, arrowheads). Stars indicate the Purkinje cell body. Scale bars indicate 20 μm
(8.65 MB TIF)

Figure S3 CHL1 Antibody Specificity (A) HEK cells transfected with CHL1 were recognized by the CHL1 peptide antibodies (A1), and nontransfected cells were not (A2).
(B) Our CHL1 peptide antibody showed no signals in the cerebellum of *CHL1^−/−^* mice.
(4.57 MB TIF)

Figure S4 Relationship among Bergmann Glial Fibers, GAD65, and CHL1 in the Molecular Layer (A) Radial BG fibers extended elaborate lateral appendages at P18. Single BG cells were labeled by electroporation at P3 to express GFP (A1), and were imaged at P18 (A2). Note the extensive lateral appendages of BG fibers. (A3) is a 3-D representation of the boxed area in (A2). Arrows indicate the lateral appendages of BG fibers.
(B) GFAP-GFP transgenic mice revealed that mature BG cells extended prominent radial fibers containing GFAP (red); these BG fibers further elaborated a extensive web of lateral appendages and fine process that are GFAP negative. Stars indicate soma of Bergmann glia; arrowheads, lateral appendages; arrows, fine BG processes.
(C and D) At P18 (C) and P21 (D), GAD65 puncta are often organized along the vertical stripe pattern of CHL1 signals (arrowheads), which colocalized with GFAP (Figure 4E). Note that CHL1 is also expressed in stellate cells (C2, stars).
(E), Occasionally, strings of GAD65 puncta were detected along the lateral appendage of BG fiber labeled by GFAP (arrows) at these ages. Scale bars indicate 20 μm.
(8.91 MB TIF)

Figure S5 Normal Parallel Fiber and Climbing Fiber Innervation in *CHL1^−/−^* Mice (A and B) At P42, climbing fiber synapses labeled by VgluT2 in WT (A) and *CHL1^−/−^* (B) mice. VgluT2 is partially and equally associated with GFAP fibers in both WT (A3, arrows) and *CHL1^−/−^* (B3, arrows) mice.
(C) Quantification of VgluT2 and GFAP association show no difference between WT and *CHL1^−/−^* mice.
(D and E) Parallel fiber synapses in the ML labeled by VgluT1 are similar in WT (D) and *CHL1^−/−^* mice (E).
(F) Mean fluorescent intensity of VgluT1 signals in the ML was the same between WT and *CHL1^−/−^* mice.
(G–K) Ultrastructural analysis revealed that neither parallel fiber (PF [I–J]) nor climbing fibers (CF [G–K]) synapses showed any discernable defects in *CHL1^−/−^* mice compared to WT littermates. (I) A climbing fiber synapse confirmed with VgluT2 immunoelectron microscopy in *CHL1^−/−^* mice. Pd, Purkinje dendrite; Sp, spine. Scale bars indicate 20 μm.
(8.53 MB TIF)

Figure S6 Developing Stellate Axons Showed Aberrant Arborization in *CHL1^−/−^* Mice (A) At P16, stellate cells in *CHL1^−/−^* mice extended their axons but failed to associate with the GFAP-labeled BG fibers (arrows).
(B and C) At more-mature ages (P20 and P40), stellate cell axons were still largely not associated with BG fibers. Note that at P40 (C), some of these stellate axons extended rather randomly, twisted, tangled, and even circled around (arrows). See Figure 2 for comparison with WT stellate axons. Scale bars indicate 20 μm.
(8.36 MB TIF)

Figure S7 Normal Basket Axon Arbor and Pinceau Synapses in *CHL1^−/−^* Mice (A) At single–basket cell resolution from PV-GFP (B20 mice), pinceau synapses (arrows) developed normally in *CHL1^−/−^* mice and expressed GAD65 (A2, arrows).
(B) Basket axons (green) grew along Purkinje proximal dendrite in *CHL1^−/−^* mice (B2–3) as in WT mice (Figure 1H).
(C and D) Ultrastructural analysis revealed similar basket synapses onto Purkinje soma in WT (C) and *CHL1^−/^* ^−^ (D) mice.
Bt, basket; Pc, Purkinje cell. Scale bars indicate 20 μm.
(8.58 MB TIF)

## Abbreviations

AIS: axon initial segment
BAC: bacterial artificial chromosome
BG: Bergmann glia
GFP: green fluorescent protein
L1CAM: L1 family immunoglobulin cell adhesion molecule
ML: molecular layer
P: postnatal day
PCL: Purkinje cell layer
Pv: parvalbumin
WT: wild-type
