# Supplementary figures and images for: Bergmann Glia and the Recognition Molecule CHL1 Organize GABAergic Axons and Direct Innervation of Purkinje Cell Dendrites
Source: PLoS Biol. 2008 Apr 29;6(4):e103. doi: 10.1371/journal.pbio.0060103 (PMC2689695; doi:10.1371/journal.pbio.0060103)

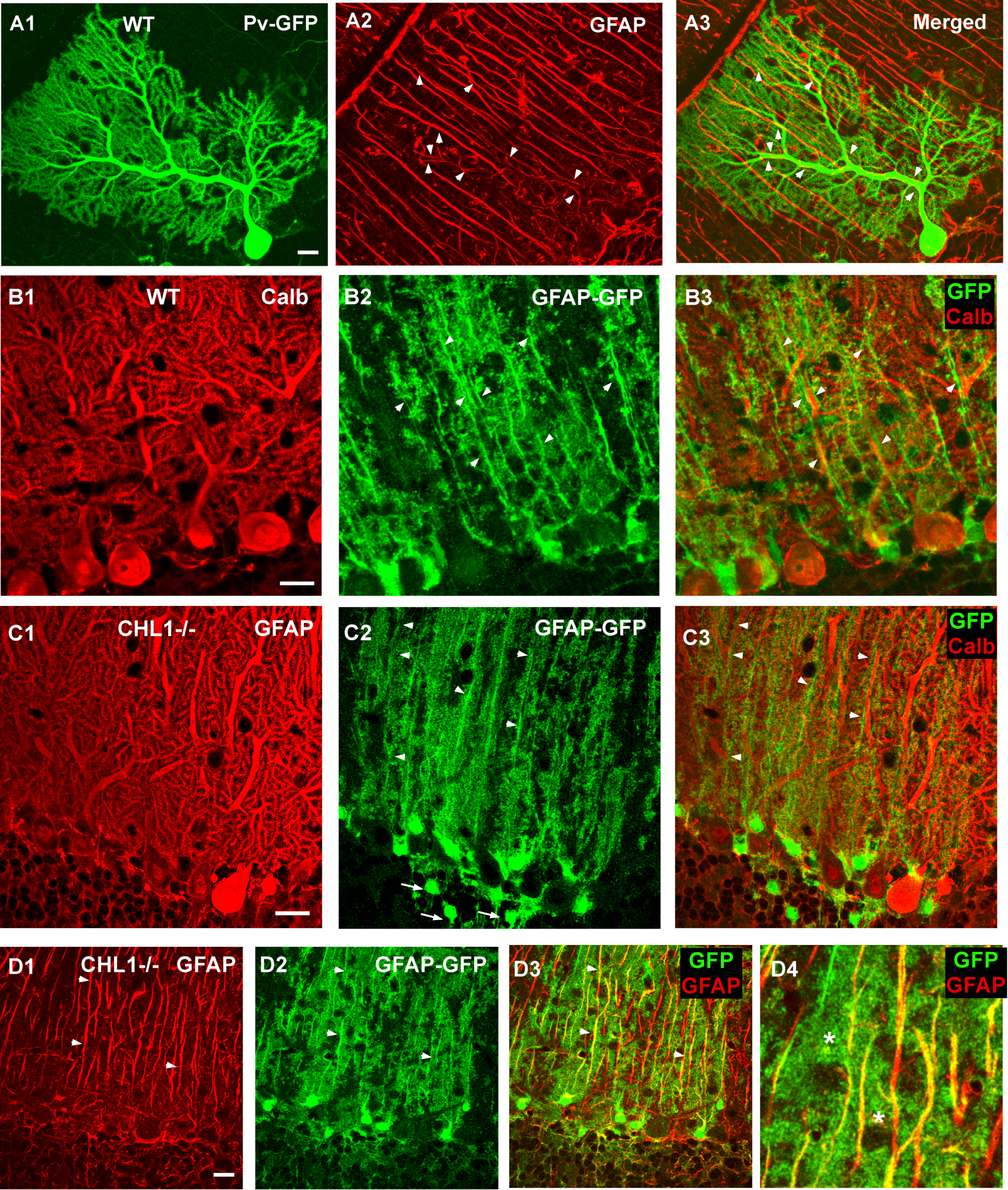

Supplement: Figure S1 — (A) A single Purkinje neuron from a PV-GFP (B20) mouse colabeled with GFAP immunofluorescence (red) revealed patchy, en passant–type, rather than extensive, association between BG fibers and Purkinje dendrite (arrowheads). (B) Complete BG fibers visualized by GFAP-GFP mice colabeled with calbindin (red) showed that a single branch of BG fiber likely impinges upon multiple intercalated Purkinje dendrites in a patchy manner (arrowheads). (C and D) In CHL1−/− mice, the GFAP fibers, BG vertical palisades (arrowheads), and lateral appendages (stars) of BG fibers all appeared similar to those in WT mice; there was occasional mispositioning of BG cell soma (C2, arrows). Scale bars indicate 20 μm. (8.72 MB TIF) [file pbio.0060103.sg001.tif]

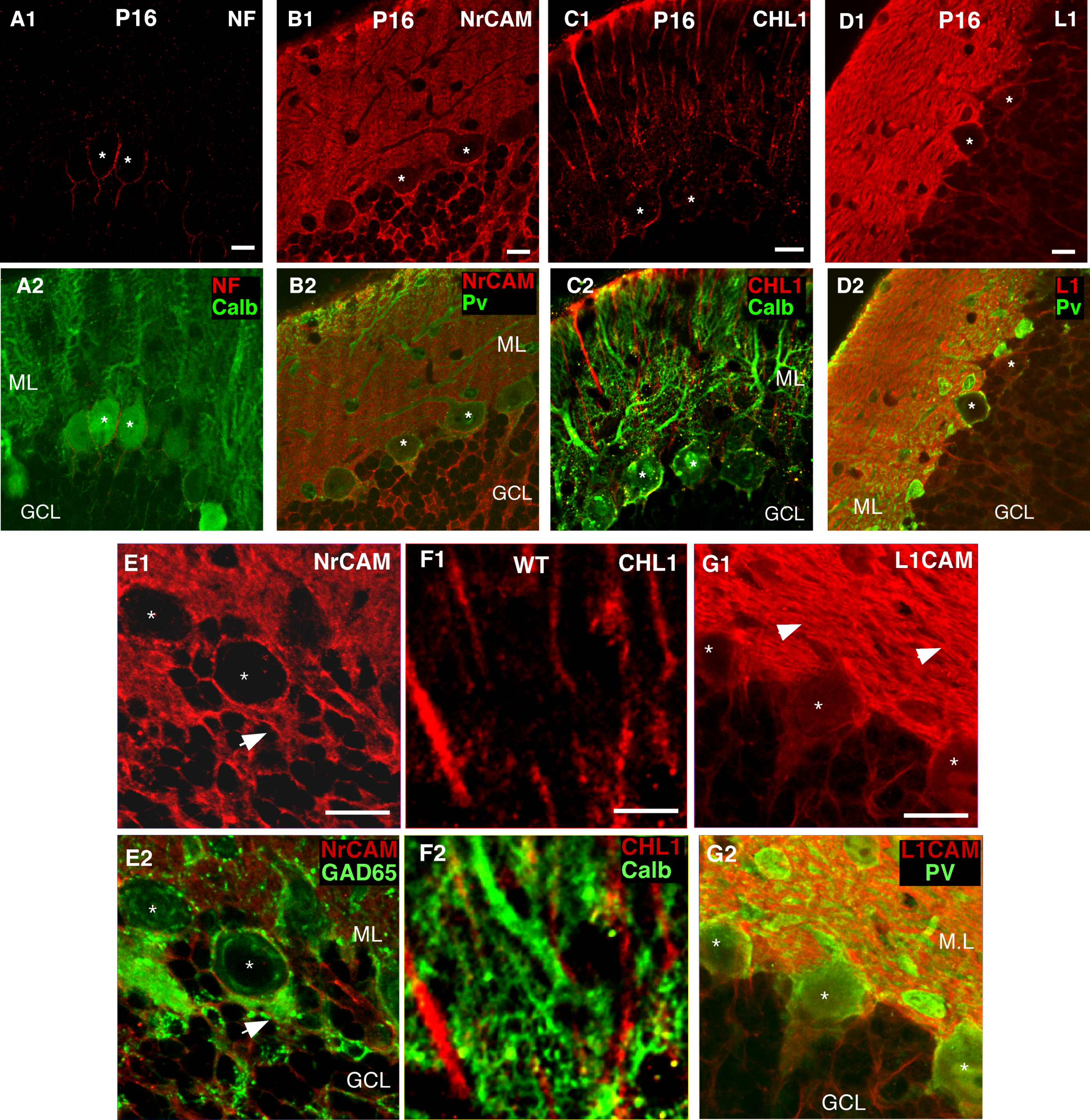

Supplement: Figure S2 — (A–D) Members of L1CAMs were differentially localized to subcellular compartments in neurons and glia cells in cerebellum at P16. (A) Neurofascin186 was highly restricted to AIS-soma of Purkinje cells. (B) NrCAM was more diffusely, but not ubiquitously, expressed in the ML. (C) CHL1 was distributed in a prominent radial stripe pattern. (D) L1 was abundantly expressed in parallel fibers and other unmyelinated and premyelinated axons. Purkinje cells were labeled by either Pv (B2 and D2) or calbindin (A2 and C2) antibodies. (E) A high-magnification view of NrCAM colabeled with GAD65 in the ML, PCL, and granule cell layer (arrows). Note that NrCAM enwrapped GAD65-positive pinceau synapses at Purkinje AIS (arrow), suggesting its localization to the basal lamellae of BG cells. (F) No coalignment of stripe patterns of CHL1 immunofluorescence (red) with Purkinje dendrite (calbindin, green). (G) L1 is prominently expressed by granule cell axons and likely other unmyelinated axons. Note the fiber-like labeling in the molecular layer (G1, arrowheads). Stars indicate the Purkinje cell body. Scale bars indicate 20 μm (8.65 MB TIF) [file pbio.0060103.sg002.tif]

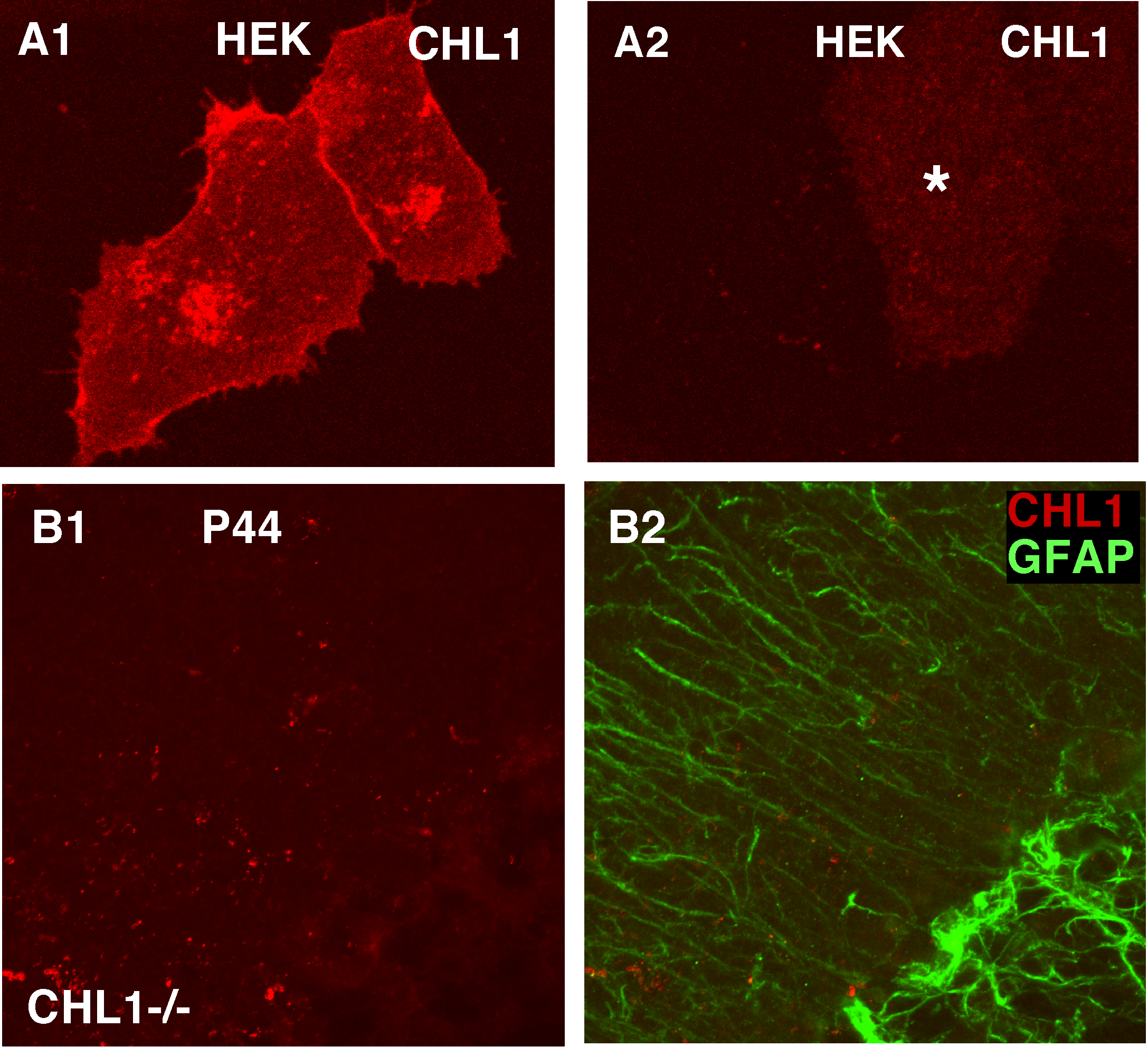

Supplement: Figure S3 — (A) HEK cells transfected with CHL1 were recognized by the CHL1 peptide antibodies (A1), and nontransfected cells were not (A2). (B) Our CHL1 peptide antibody showed no signals in the cerebellum of CHL1−/− mice. (4.57 MB TIF) [file pbio.0060103.sg003.tif]

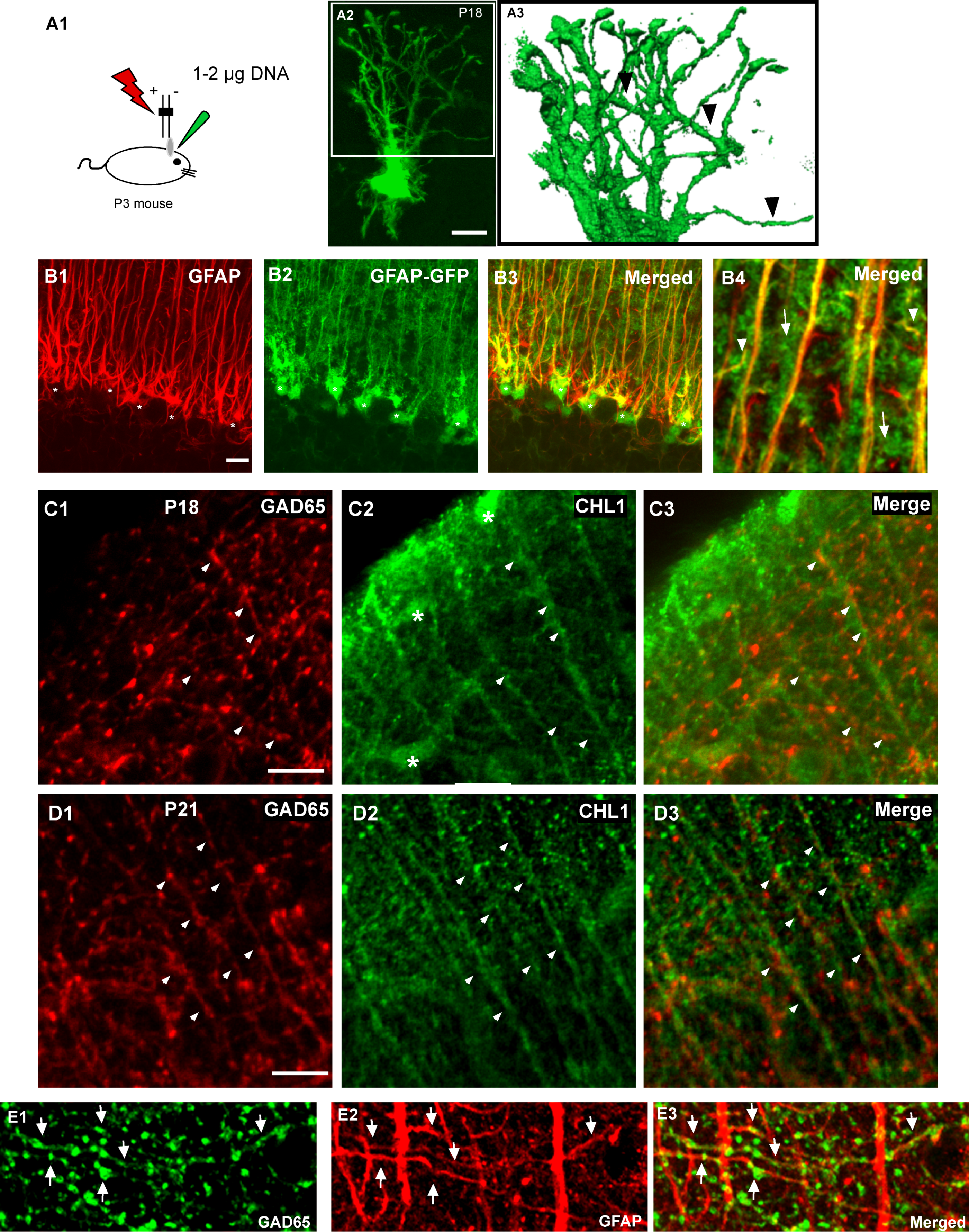

Supplement: Figure S4 — (A) Radial BG fibers extended elaborate lateral appendages at P18. Single BG cells were labeled by electroporation at P3 to express GFP (A1), and were imaged at P18 (A2). Note the extensive lateral appendages of BG fibers. (A3) is a 3-D representation of the boxed area in (A2). Arrows indicate the lateral appendages of BG fibers. (B) GFAP-GFP transgenic mice revealed that mature BG cells extended prominent radial fibers containing GFAP (red); these BG fibers further elaborated a extensive web of lateral appendages and fine process that are GFAP negative. Stars indicate soma of Bergmann glia; arrowheads, lateral appendages; arrows, fine BG processes. (C and D) At P18 (C) and P21 (D), GAD65 puncta are often organized along the vertical stripe pattern of CHL1 signals (arrowheads), which colocalized with GFAP (Figure 4E). Note that CHL1 is also expressed in stellate cells (C2, stars). (E), Occasionally, strings of GAD65 puncta were detected along the lateral appendage of BG fiber labeled by GFAP (arrows) at these ages. Scale bars indicate 20 μm. (8.91 MB TIF) [file pbio.0060103.sg004.tif]

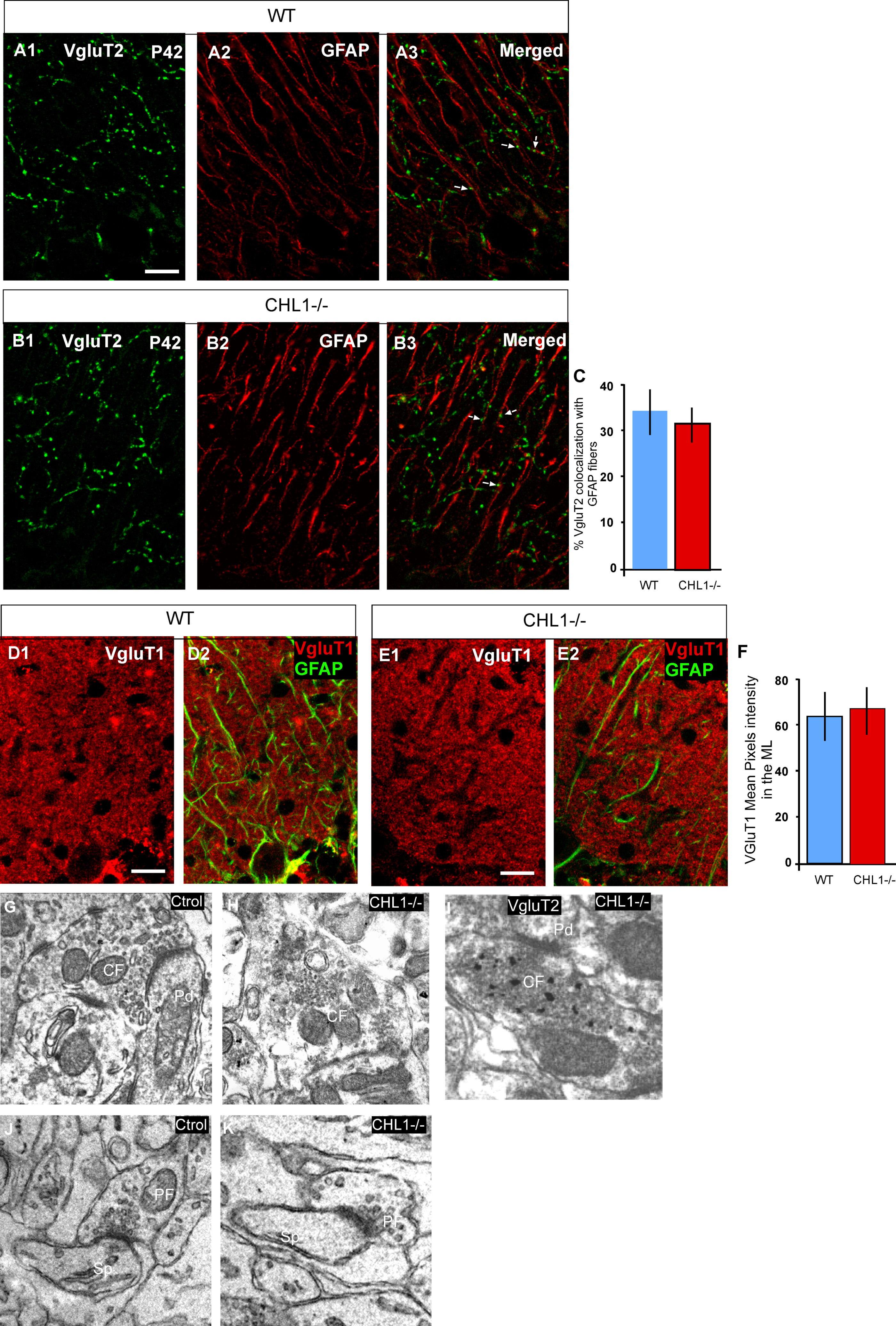

Supplement: Figure S5 — (A and B) At P42, climbing fiber synapses labeled by VgluT2 in WT (A) and CHL1−/− (B) mice. VgluT2 is partially and equally associated with GFAP fibers in both WT (A3, arrows) and CHL1−/− (B3, arrows) mice. (C) Quantification of VgluT2 and GFAP association show no difference between WT and CHL1−/− mice. (D and E) Parallel fiber synapses in the ML labeled by VgluT1 are similar in WT (D) and CHL1−/− mice (E). (F) Mean fluorescent intensity of VgluT1 signals in the ML was the same between WT and CHL1−/− mice. (G–K) Ultrastructural analysis revealed that neither parallel fiber (PF [I–J]) nor climbing fibers (CF [G–K]) synapses showed any discernable defects in CHL1−/− mice compared to WT littermates. (I) A climbing fiber synapse confirmed with VgluT2 immunoelectron microscopy in CHL1−/− mice. Pd, Purkinje dendrite; Sp, spine. Scale bars indicate 20 μm. (8.53 MB TIF) [file pbio.0060103.sg005.tif]

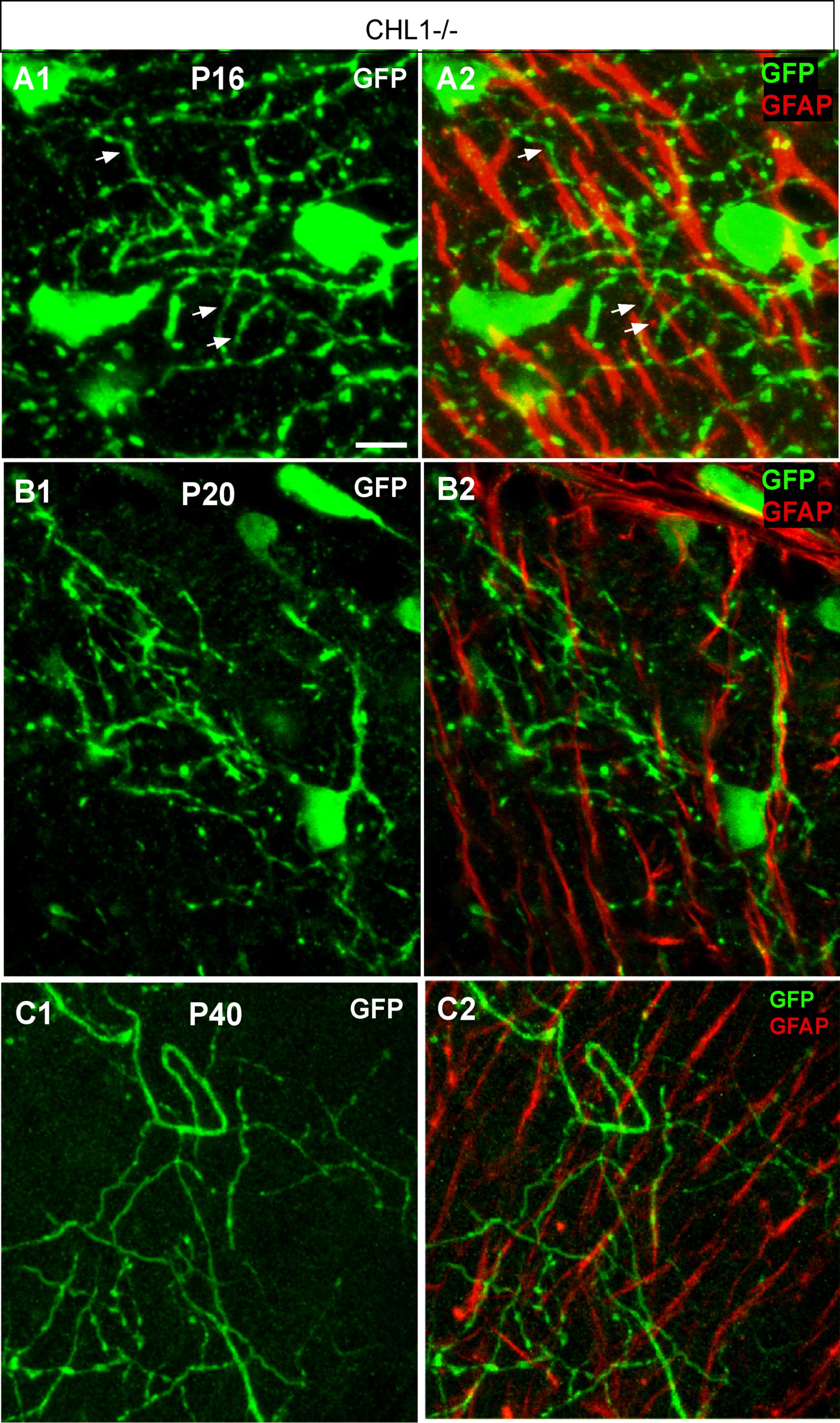

Supplement: Figure S6 — (A) At P16, stellate cells in CHL1−/− mice extended their axons but failed to associate with the GFAP-labeled BG fibers (arrows). (B and C) At more-mature ages (P20 and P40), stellate cell axons were still largely not associated with BG fibers. Note that at P40 (C), some of these stellate axons extended rather randomly, twisted, tangled, and even circled around (arrows). See Figure 2 for comparison with WT stellate axons. Scale bars indicate 20 μm. (8.36 MB TIF) [file pbio.0060103.sg006.tif]

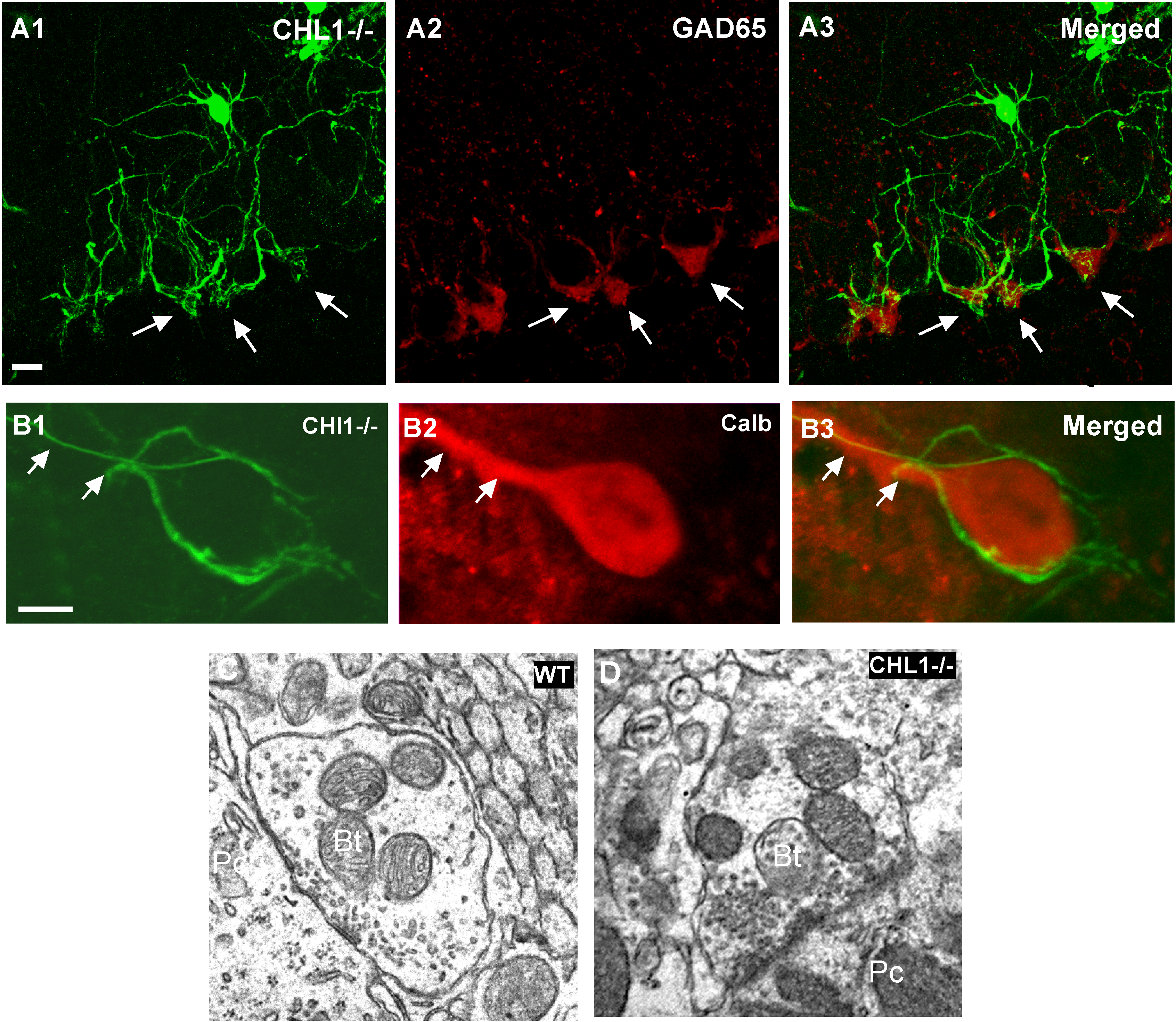

Supplement: Figure S7 — (A) At single–basket cell resolution from PV-GFP (B20 mice), pinceau synapses (arrows) developed normally in CHL1−/− mice and expressed GAD65 (A2, arrows). (B) Basket axons (green) grew along Purkinje proximal dendrite in CHL1−/− mice (B2–3) as in WT mice (Figure 1H). (C and D) Ultrastructural analysis revealed similar basket synapses onto Purkinje soma in WT (C) and CHL1−/ − (D) mice. Bt, basket; Pc, Purkinje cell. Scale bars indicate 20 μm. (8.58 MB TIF) [file pbio.0060103.sg007.tif]
